# Supplementary material for: Dual protection by Bcp1 and Rkm1 ensures incorporation of uL14 into pre-60S ribosomal subunits
Source: J Cell Biol. 2024 Jul 15;223(8):e202306117. doi: 10.1083/jcb.202306117 (PMC11248248; doi:10.1083/jcb.202306117)
Supplement: Table S1 — shows yeast strains used in this study. [file JCB_202306117_TableS1.docx]

**Table S1. Yeast strains used in this study**

| **Strain #** | **genotype** | **Source** |
| --- | --- | --- |
| BY4741 | *MATa his3*Δ*1 leu2*Δ*0 met15*Δ*0 ura3*Δ*0* | Open Biosystems |
| KLY181 | *MATα his3*Δ*1 leu2*Δ*0 met15*Δ*0 ura3*Δ*0 bcp1∆::NatMX carrying bcp1ts (URA3 CEN)* | This study |
| KLY278 | *MATa his3*Δ*1 leu2*Δ*0 met15*Δ*0 ura3*Δ*0 rkm1*Δ*::KanMX* | This study |
| KLY464 | *his3*Δ*1 leu2*Δ*0 met15*Δ*0 ura3*Δ*0 rpl23a*Δ*::KanMX rpl23B*Δ*::KanMX carrying GAL::RPL23A plasmid (URA3 CEN)* | ([Ting et al., 2017](file:///D:\Kaiyin\LAB\Manuscript%20of%20Bcp1%20Rkm1%20and%20Rpl23\Manuscript\20230526_Manuscript%20of%20Bcp1.docx#_ENREF_59)) |
| KLY751 | *MATa his3*Δ*1 leu2*Δ*0 met15*Δ*0 ura3*Δ*0 RKM1-TAP::HIS3MX* | This study |
| KLY825 | *MATa his3*Δ*1 leu2*Δ*0 met15*Δ*0 ura3*Δ*0 bcp1*Δ*::NatMX rkm1*Δ*::KanMX carrying pRS415 bcp1ts (URA CEN)* | This study |
