# Supplementary material for: Dual protection by Bcp1 and Rkm1 ensures incorporation of uL14 into pre-60S ribosomal subunits
Source: J Cell Biol. 2024 Jul 15;223(8):e202306117. doi: 10.1083/jcb.202306117 (PMC11248248; doi:10.1083/jcb.202306117)
Supplement: Table S2 — lists plasmids used in this study. [file JCB_202306117_TableS2.docx]

**Table S2. Plasmids used in this study.**

| **Plasmid** | **Gene** | **Vector** | **Source** |
| --- | --- | --- | --- |
| *pGEX-4T3* |  | *Amp* |  |
| PAJ1010 | *TIF6-myc* | *CEN LEU2* | A.W. Johnson |
| PKL105 | *BCP1-myc* | *CEN LEU2* | ([Ting et al., 2017](file:///D:\Kaiyin\LAB\Manuscript%20of%20Bcp1%20Rkm1%20and%20Rpl23\Manuscript\20230526_Manuscript%20of%20Bcp1.docx#_ENREF_59)) |
| PKL112 | *bcp1△N40-GFP* | *CEN URA3* | This study |
| PKL194 | *RPL23* | *2μ HIS3* | This study |
| PKL343 | *BCP1-GFP* | *CEN URA3* | This study |
| PKL346 | *RPL23* | *pET21* | ([Ting et al., 2017](file:///D:\Kaiyin\LAB\Manuscript%20of%20Bcp1%20Rkm1%20and%20Rpl23\Manuscript\20230526_Manuscript%20of%20Bcp1.docx#_ENREF_59)) |
| PKL502 | *BCP1-His6* | *pET21* | ([Ting et al., 2017](file:///D:\Kaiyin\LAB\Manuscript%20of%20Bcp1%20Rkm1%20and%20Rpl23\Manuscript\20230526_Manuscript%20of%20Bcp1.docx#_ENREF_59)) |
| PKL519 | *GST-RKM1* | *pGEX-4T3* | This study |
| PKL522 | *RKM1* | *2μ HIS3* | This study |
| PKL531 | Rkm1-myc | *CEN LEU2* | This study |
| PKL534 | Rkm1-GFP | *CEN LEU2* | This study |
| PKL538 | *bcp1*∆*N40-His6* | *pET21* | This study |
| PKL543 | *RKM1-His6* | *pET28* | This study |
| PKL547 | *GST-KAP123* | *pGEX-4T3* | ([Ting et al., 2017](file:///D:\Kaiyin\LAB\Manuscript%20of%20Bcp1%20Rkm1%20and%20Rpl23\Manuscript\20230526_Manuscript%20of%20Bcp1.docx#_ENREF_59)) |
| PKL556 | *GST-KAP121* | *pGEX-4T3* | ([Ting et al., 2017](file:///D:\Kaiyin\LAB\Manuscript%20of%20Bcp1%20Rkm1%20and%20Rpl23\Manuscript\20230526_Manuscript%20of%20Bcp1.docx#_ENREF_59)) |
| PKL566 | *bcp1∆N10-GFP* | *CEN URA3* | This study |
| PKL567 | *bcp1N20-GFP* | *CEN URA3* | This study |
| PKL594 | *rpl23A*Δl*oop* | *pET21* | This study |
| PKL597 | *bcp1∆N10-His6* | *pET21* | This study |
| PKL598 | *bcp1∆N20-His6* | *pET21* | This study |
| PKL666 | *rpl23K106R* | *pET21* | This study |
| PKL667 | *rpl23K110R* | *pET21* | This study |
| PKL668 | *rpl23K106,110R* | *pET21* | This study |
| PKL671 | *GST-rkm1(Y273F)* | *pGEX-4T3* | This study |
| PKL710 | *RKM1-HA* | *CEN HIS3* | This study |
| PKL711 | *rkm1Y273F-HA* | *CEN HIS3* | This study |
| PKL914 | *rpl23A*Δ*loop-HA* | *CEN HIS3* | This study |
| PKL917 | *rpl23A RA-HA* | *CEN HIS3* | This study |
