# Supplementary figures and images for: Dual protection by Bcp1 and Rkm1 ensures incorporation of uL14 into pre-60S ribosomal subunits
Source: J Cell Biol. 2024 Jul 15;223(8):e202306117. doi: 10.1083/jcb.202306117 (PMC11248248; doi:10.1083/jcb.202306117)

Fig 1B

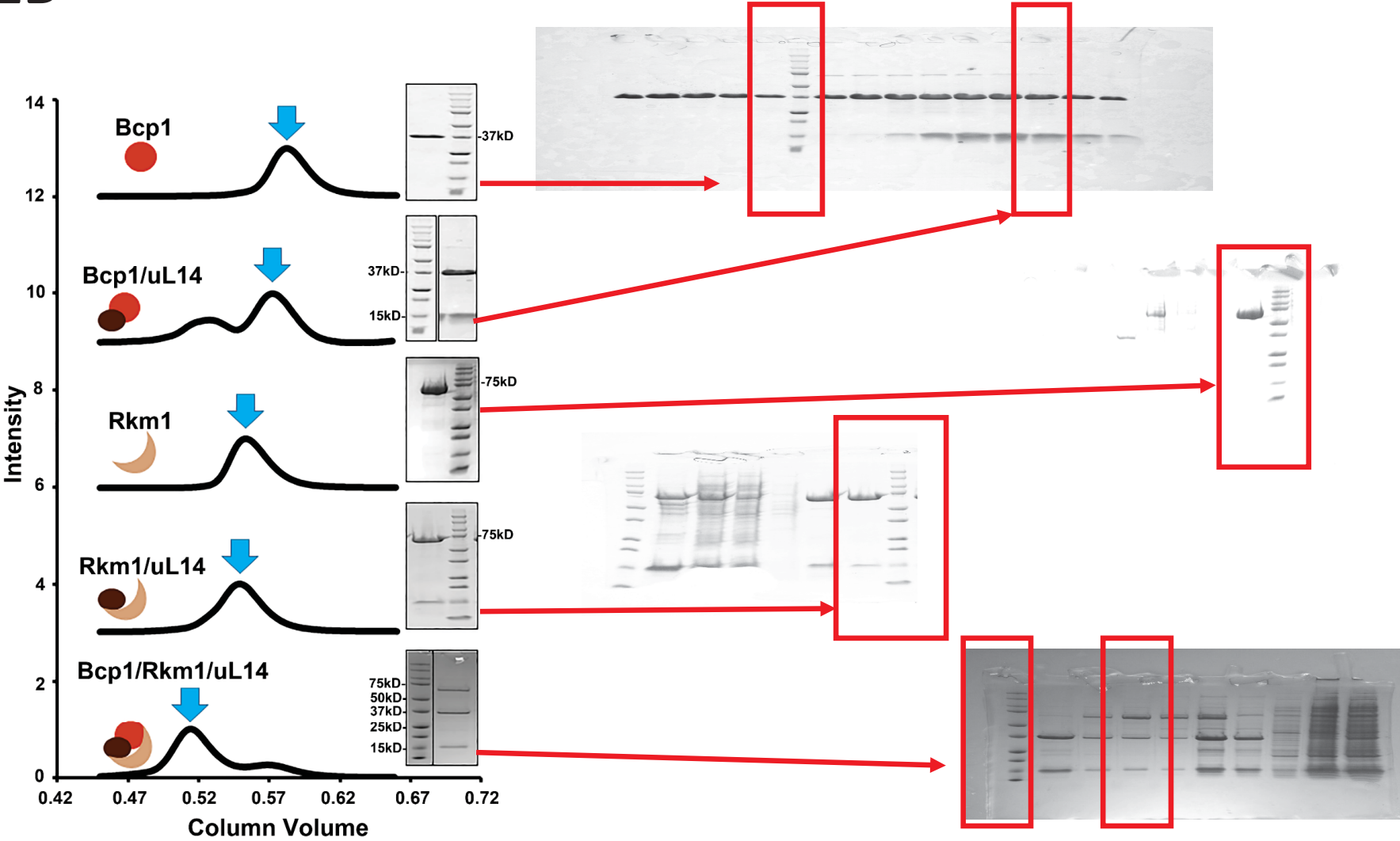

Fig 1C

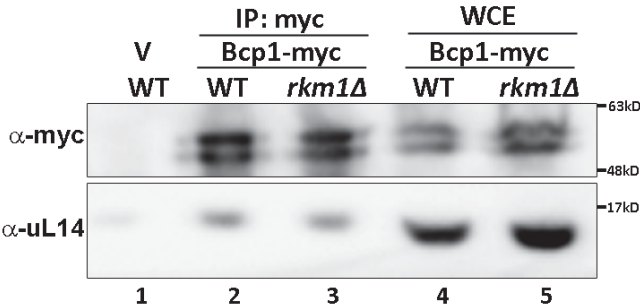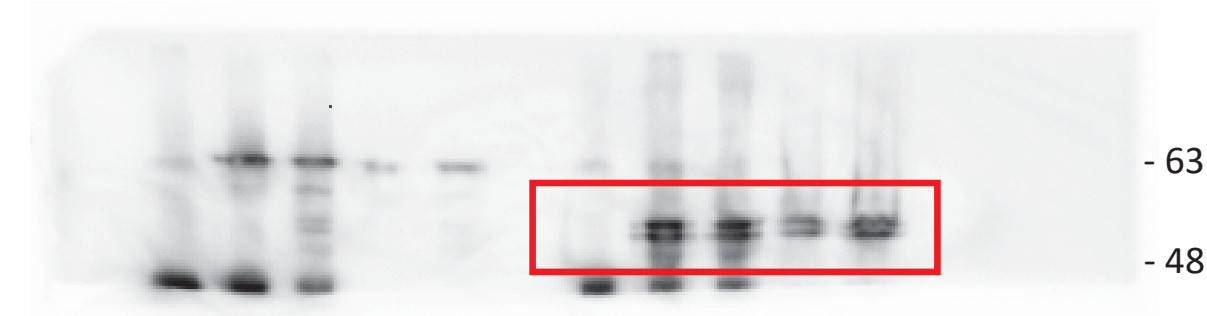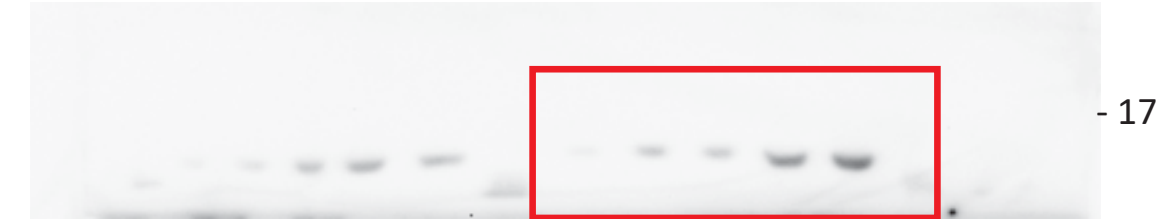

Fig 1D

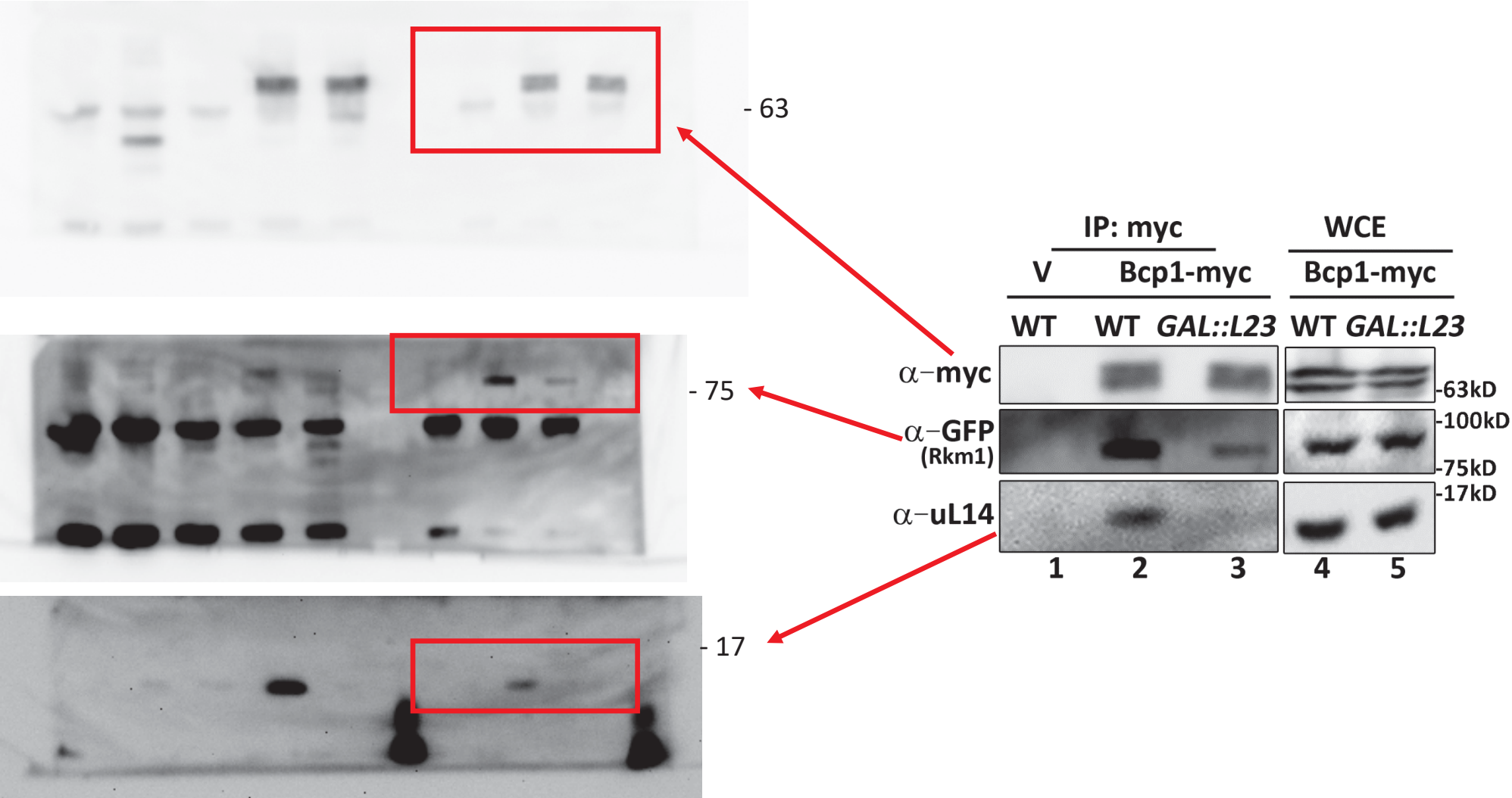

Fig 1D

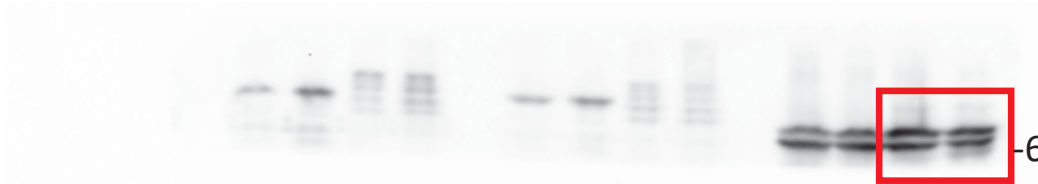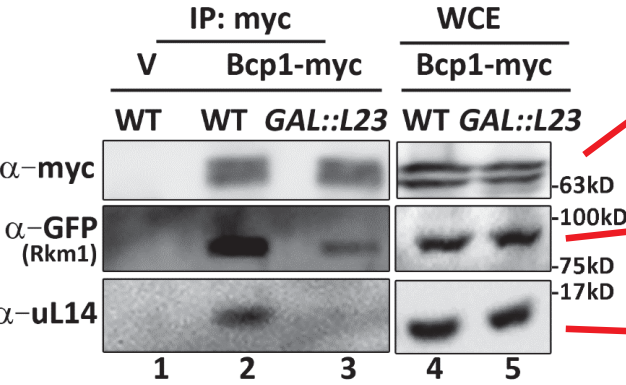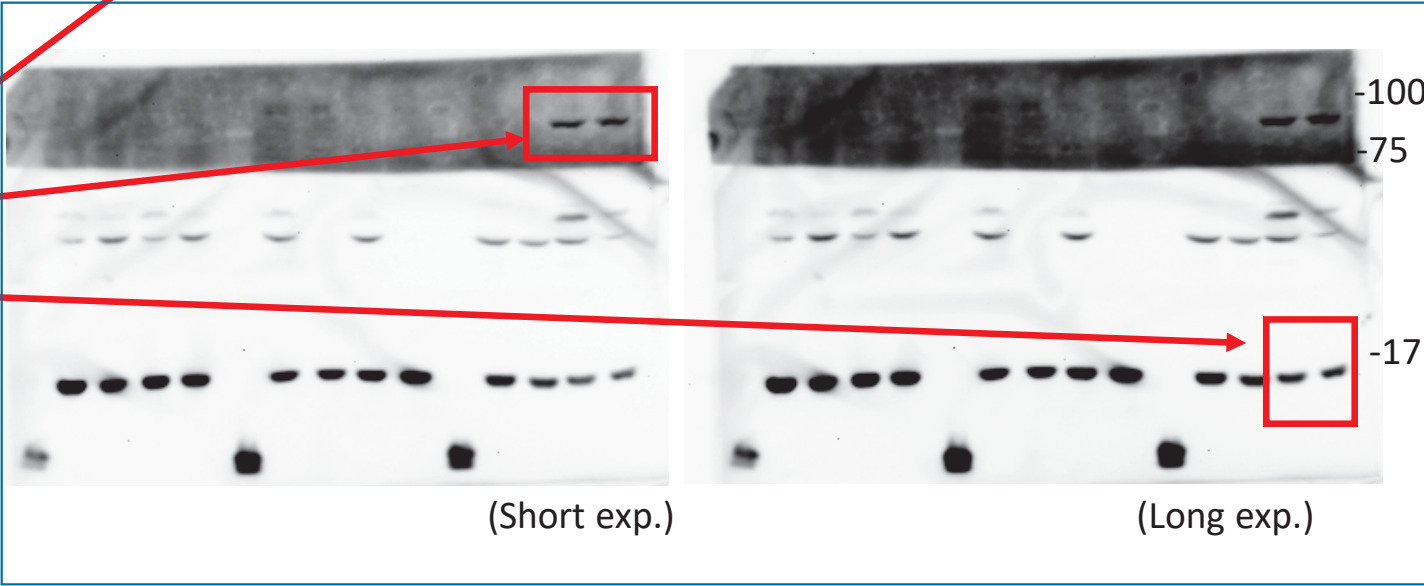

Fig 1E

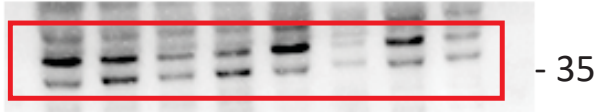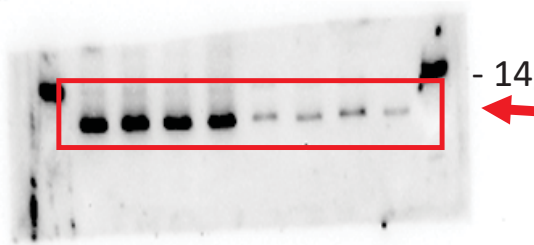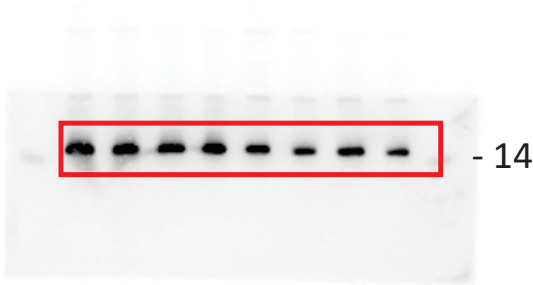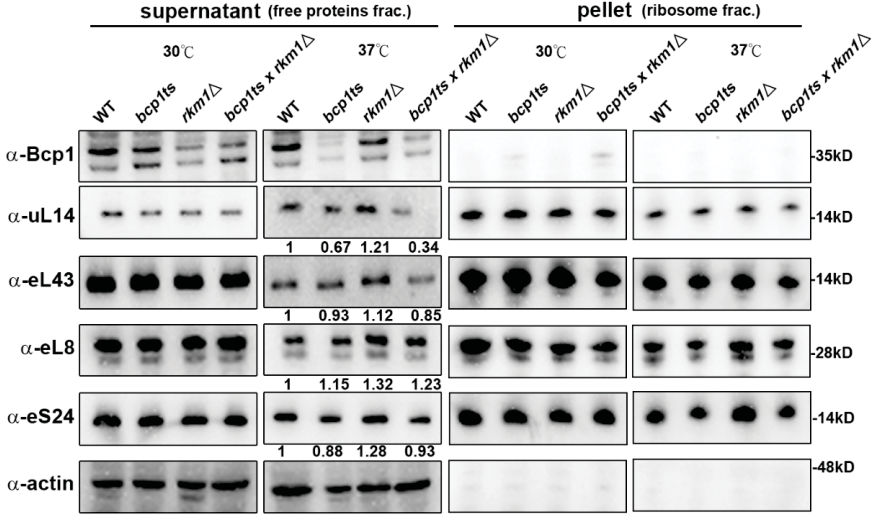

Fig 1E

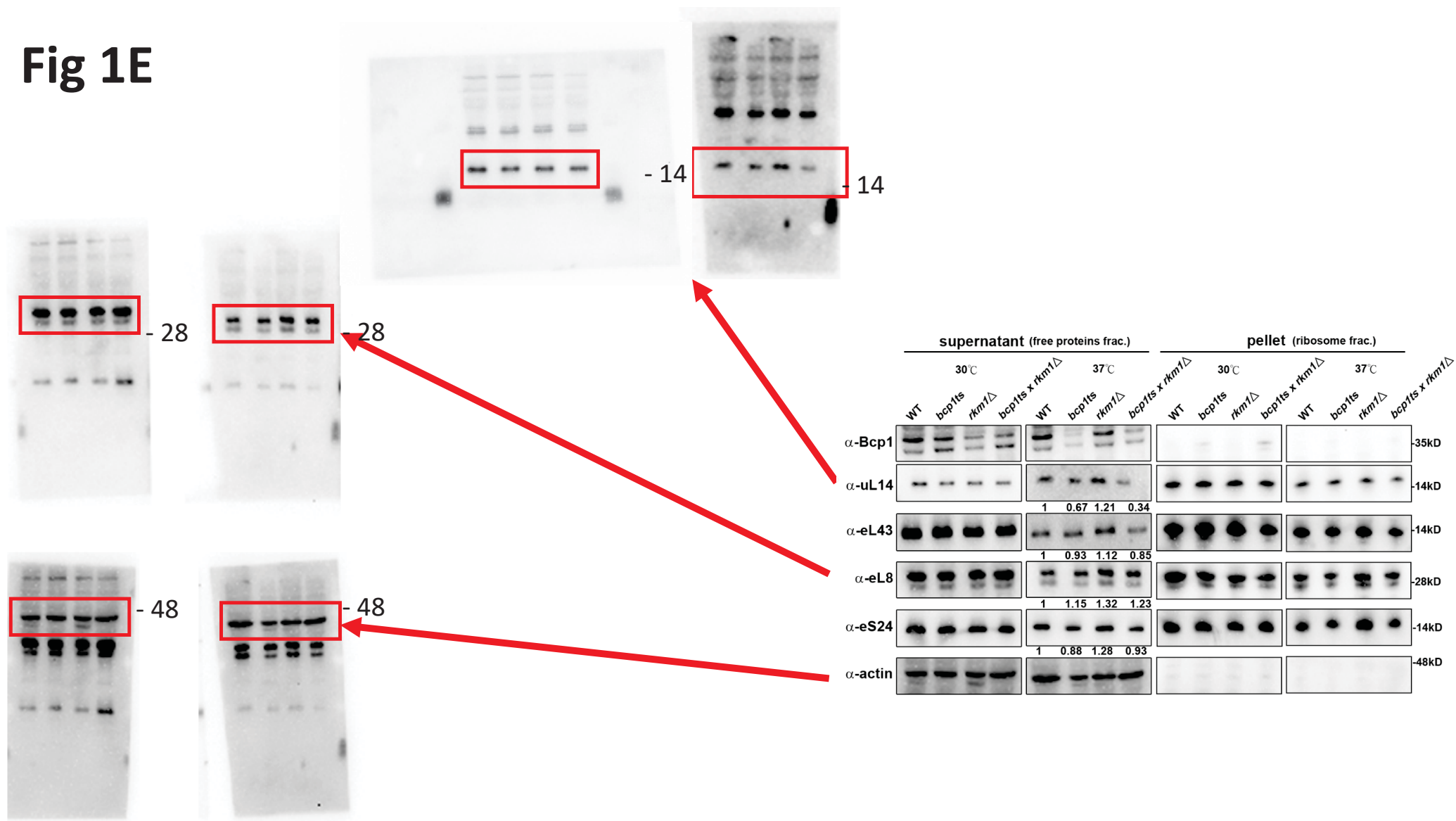

Fig 1E

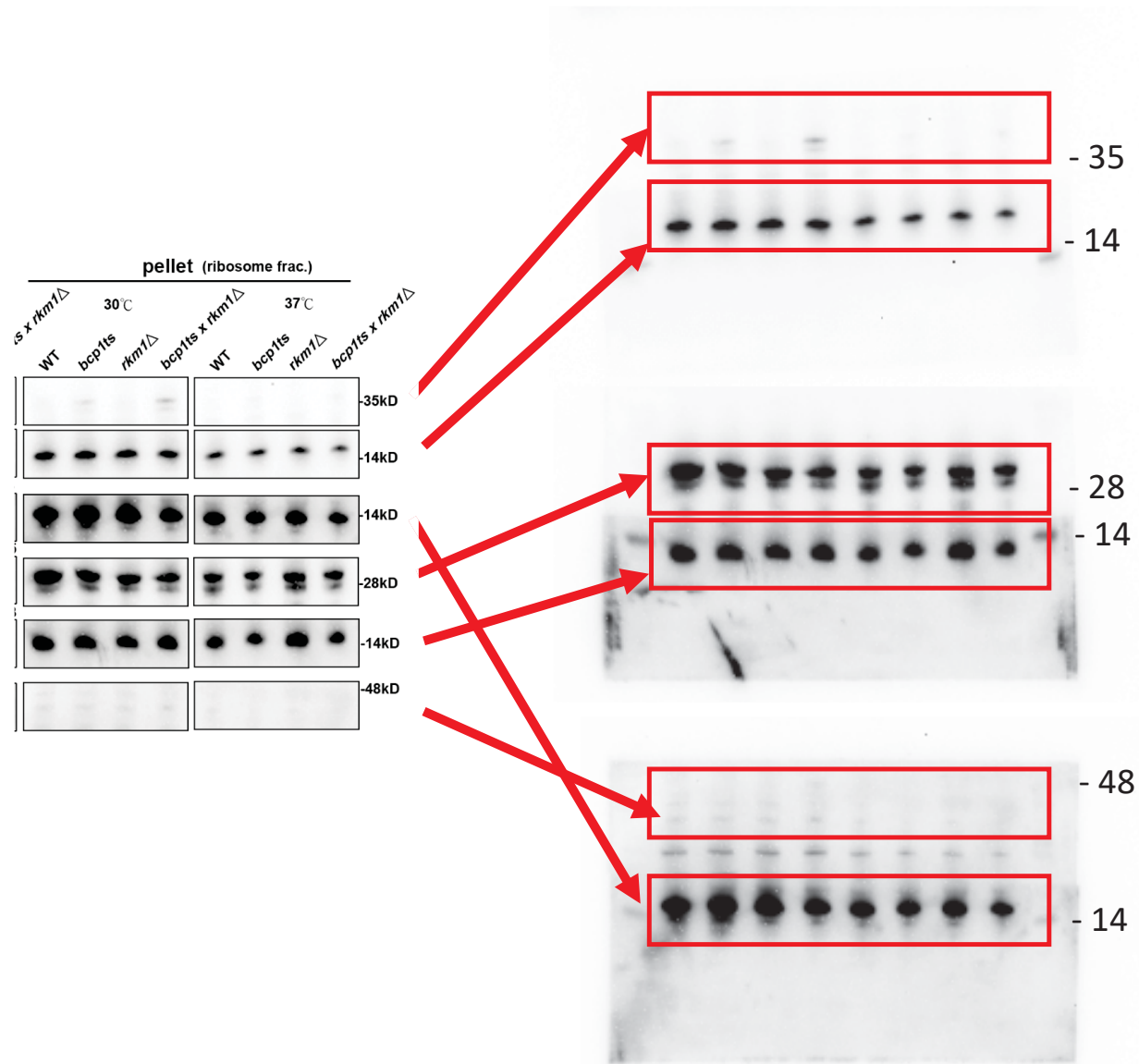

Fig 1F

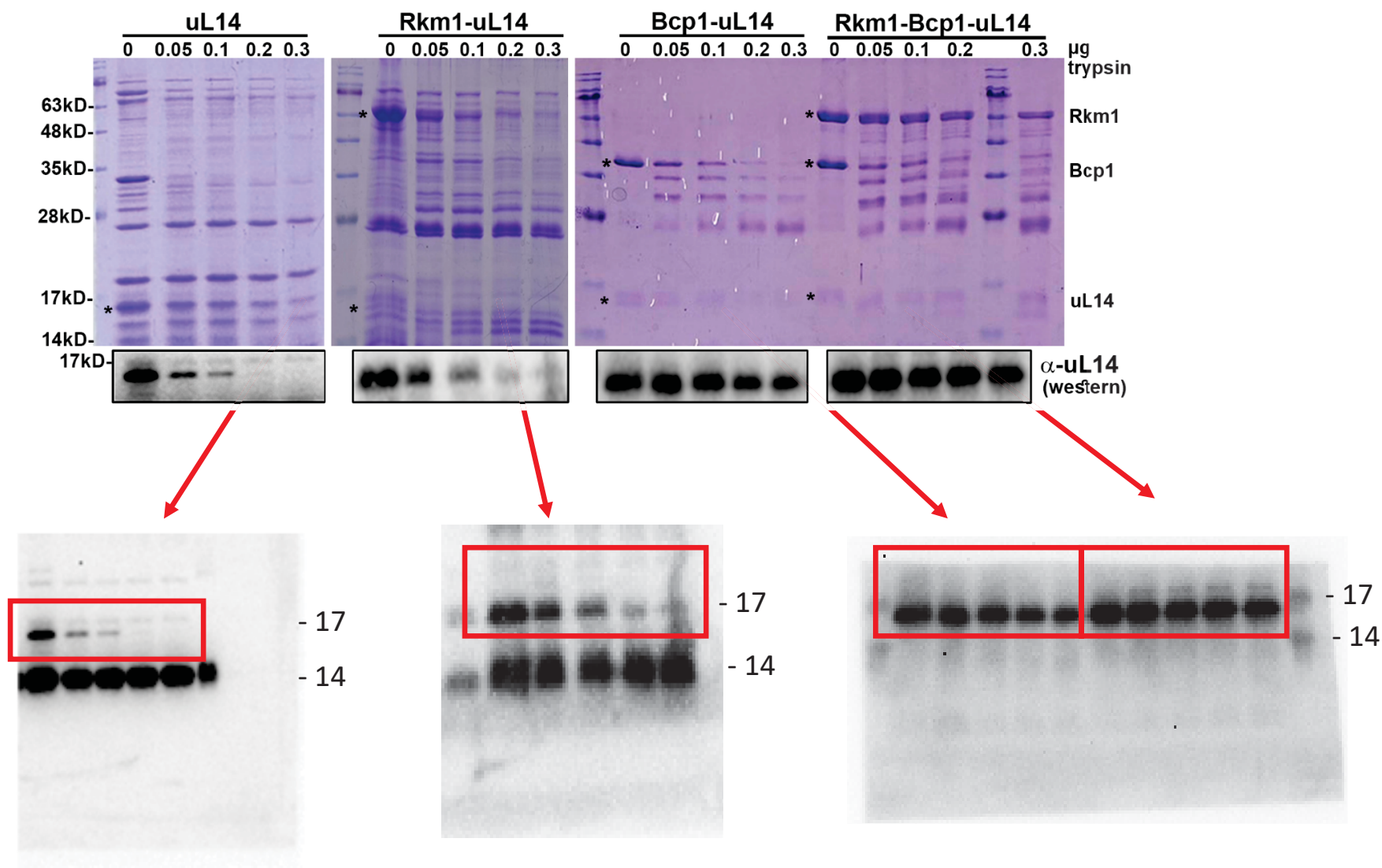

Supplement: SourceData F1 — is the source file for Fig. 1. [file JCB_202306117_SourceDataF1.pdf]

Fig 2B

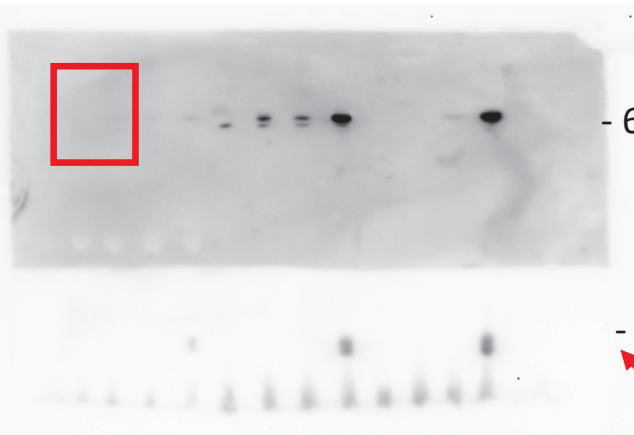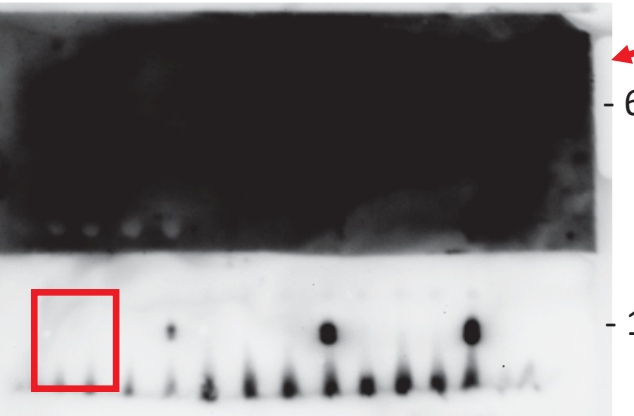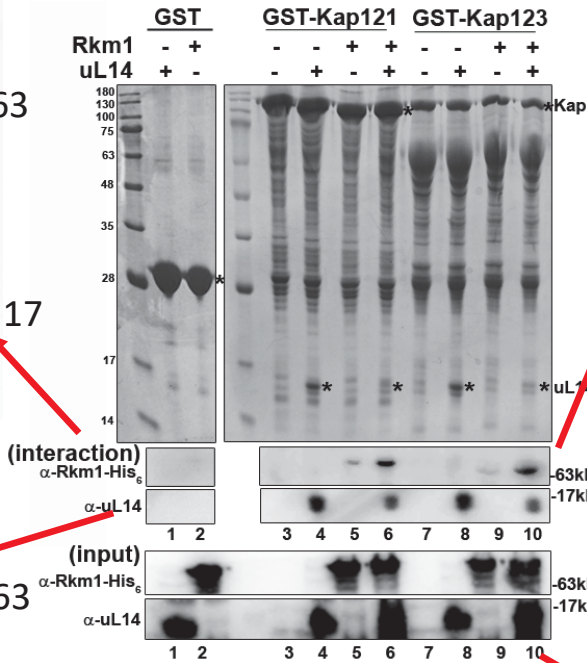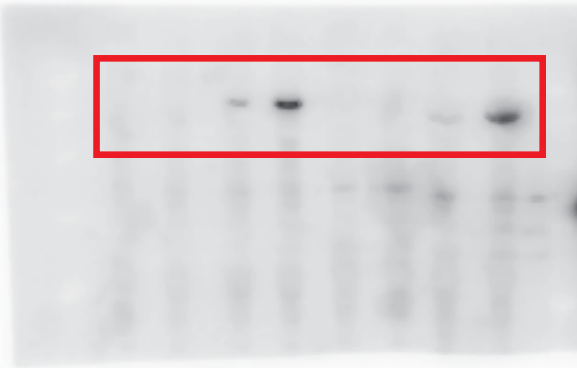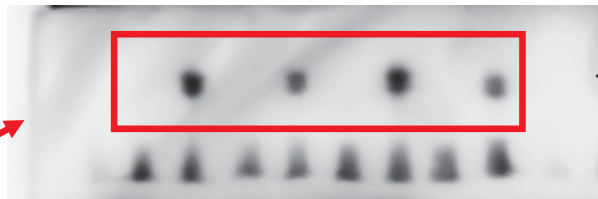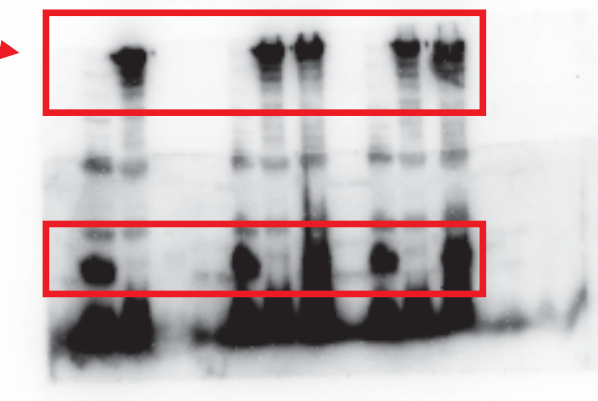

Fig 2E

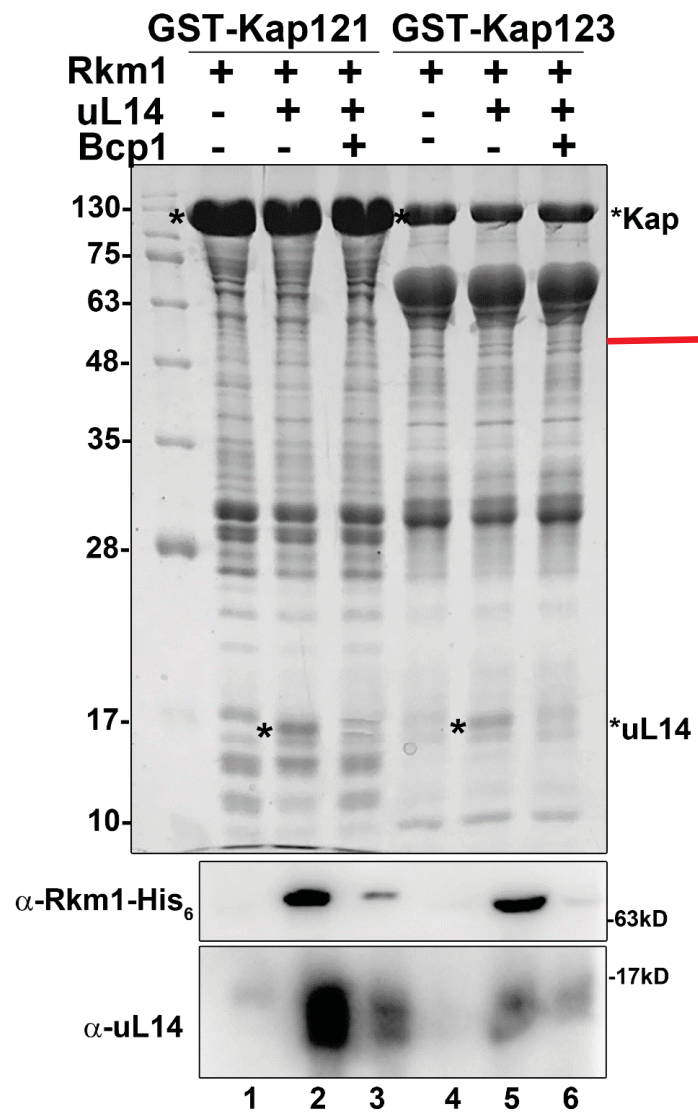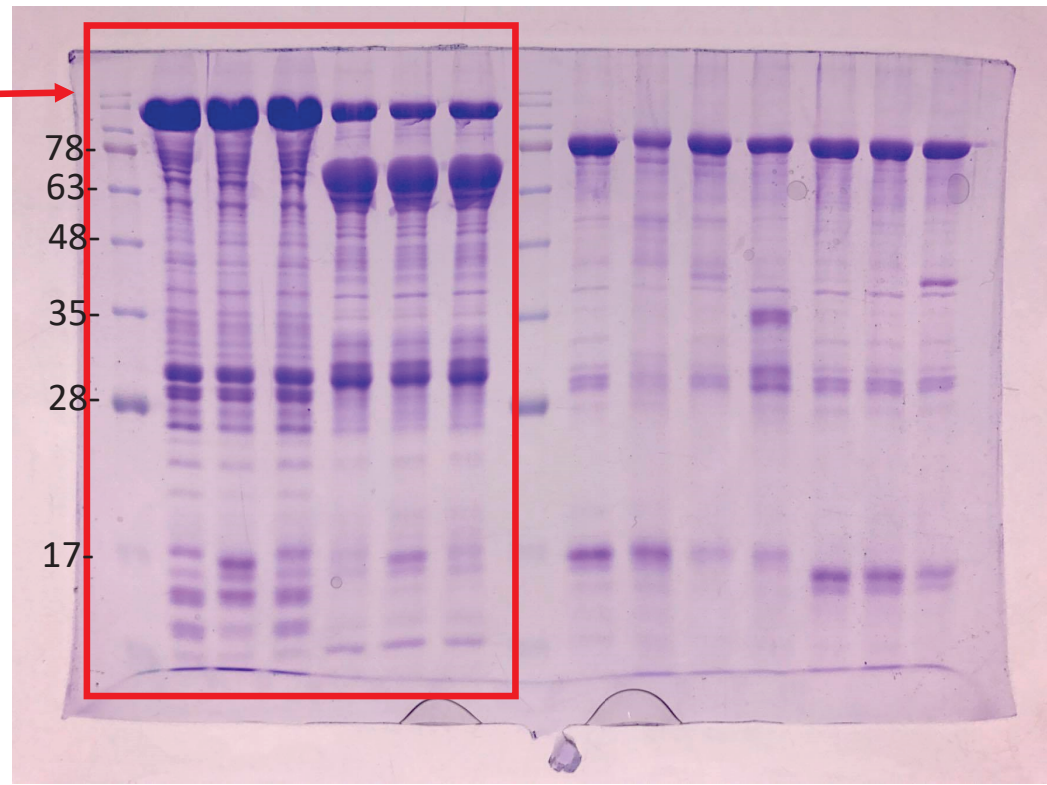

Fig 2E

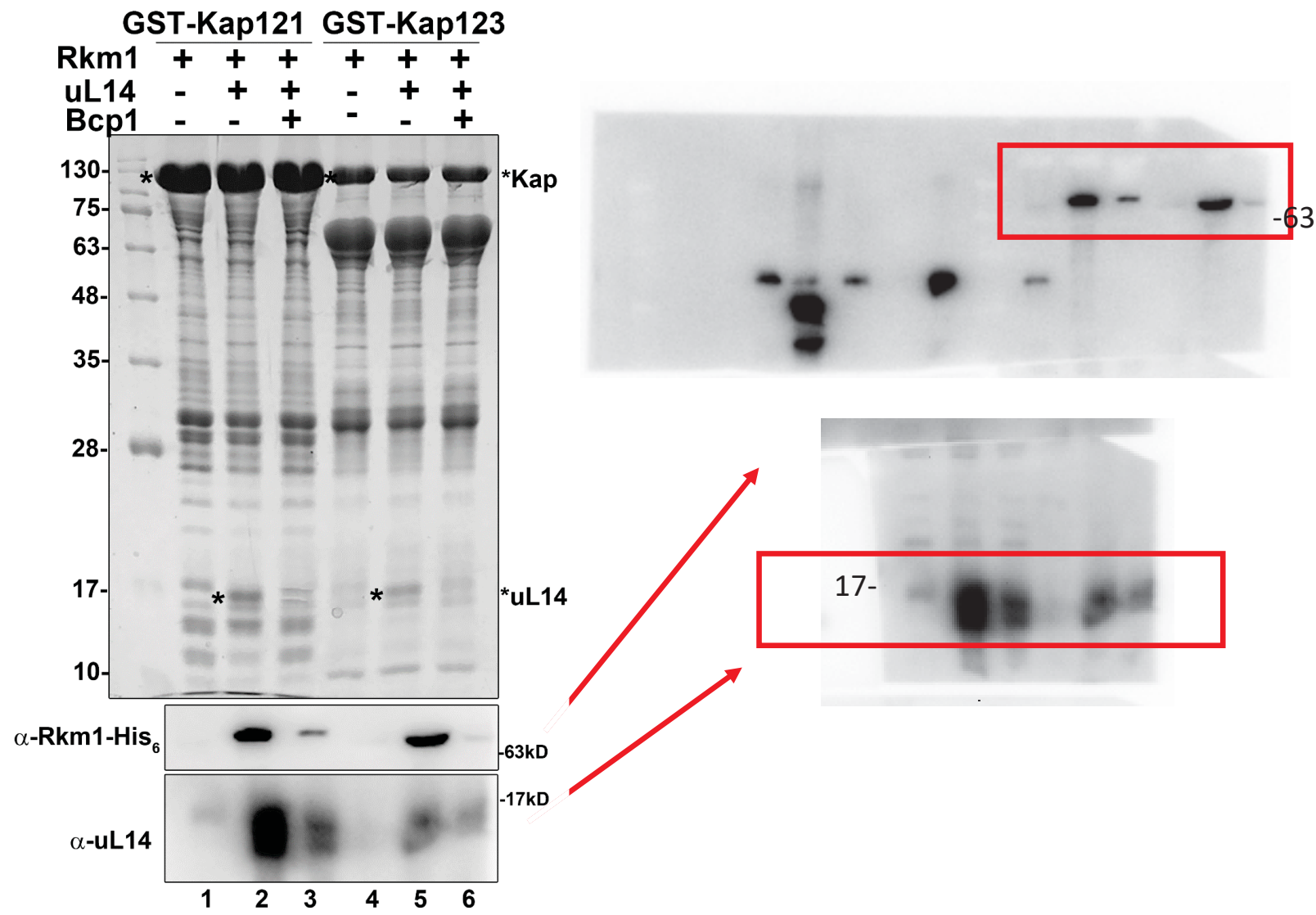

Fig 2F

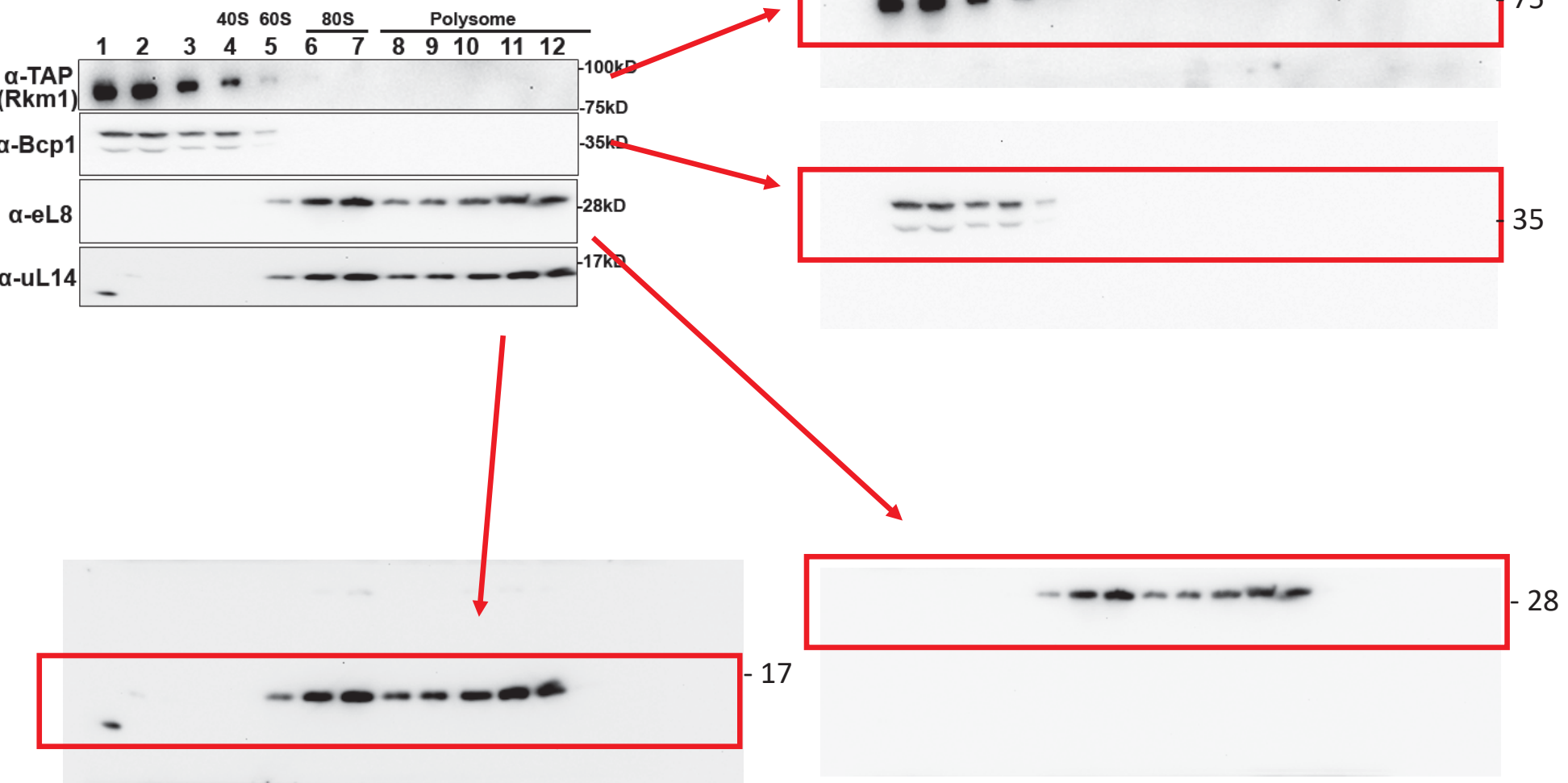

Supplement: SourceData F2 — is the source file for Fig. 2. [file JCB_202306117_SourceDataF2.pdf]

**Fig 4C**

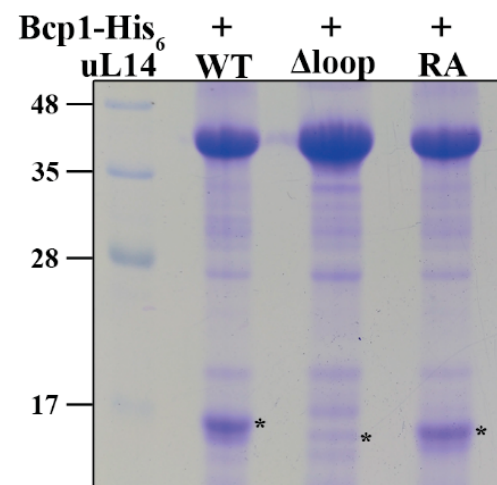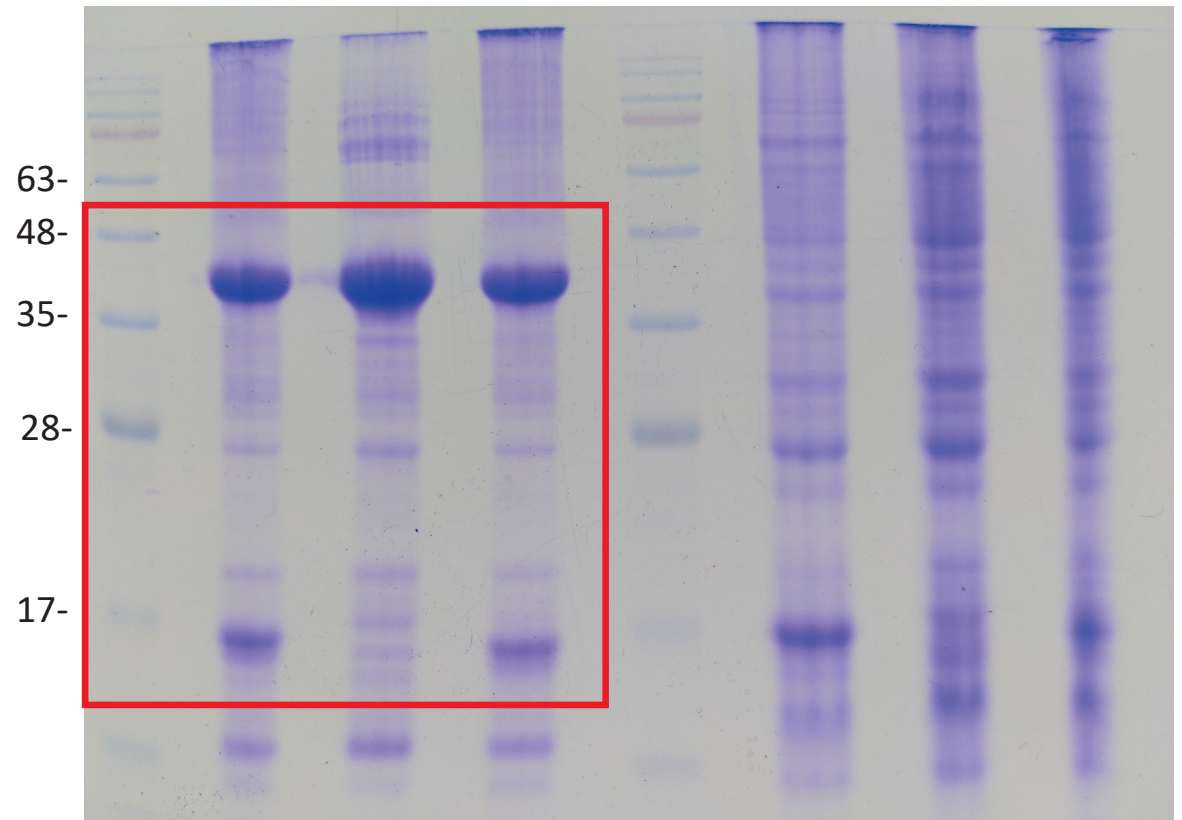

Fig 4D

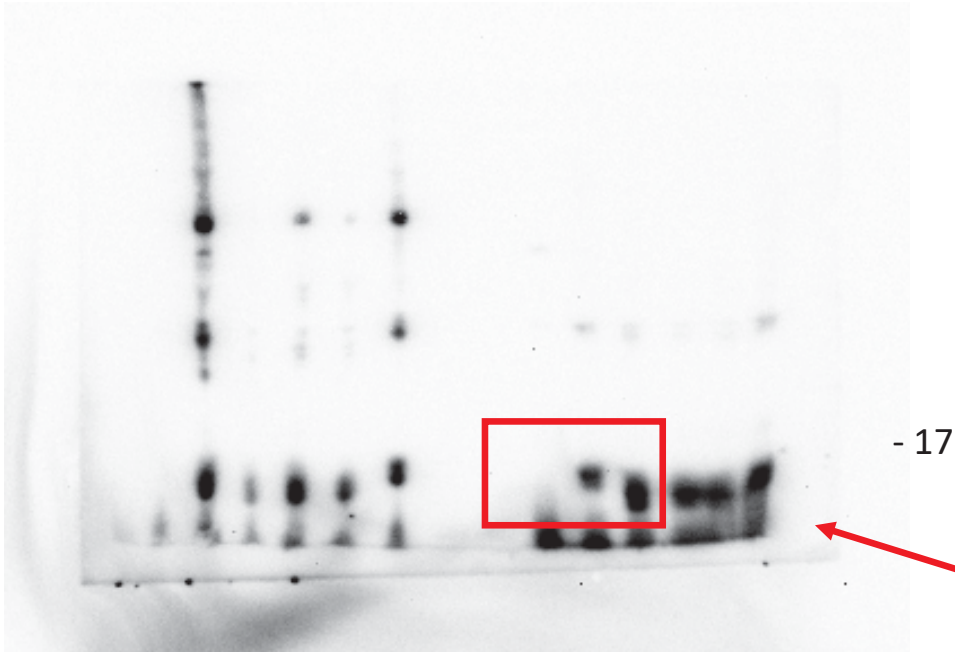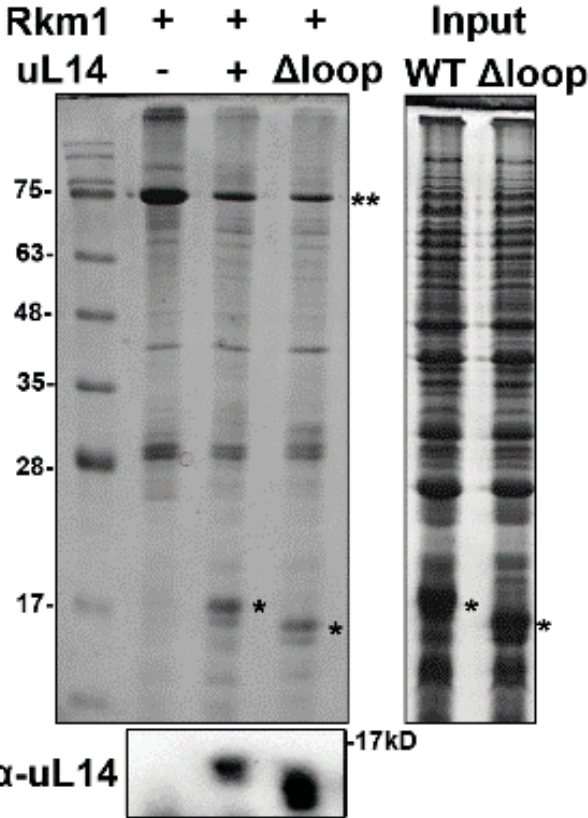

Fig 4E

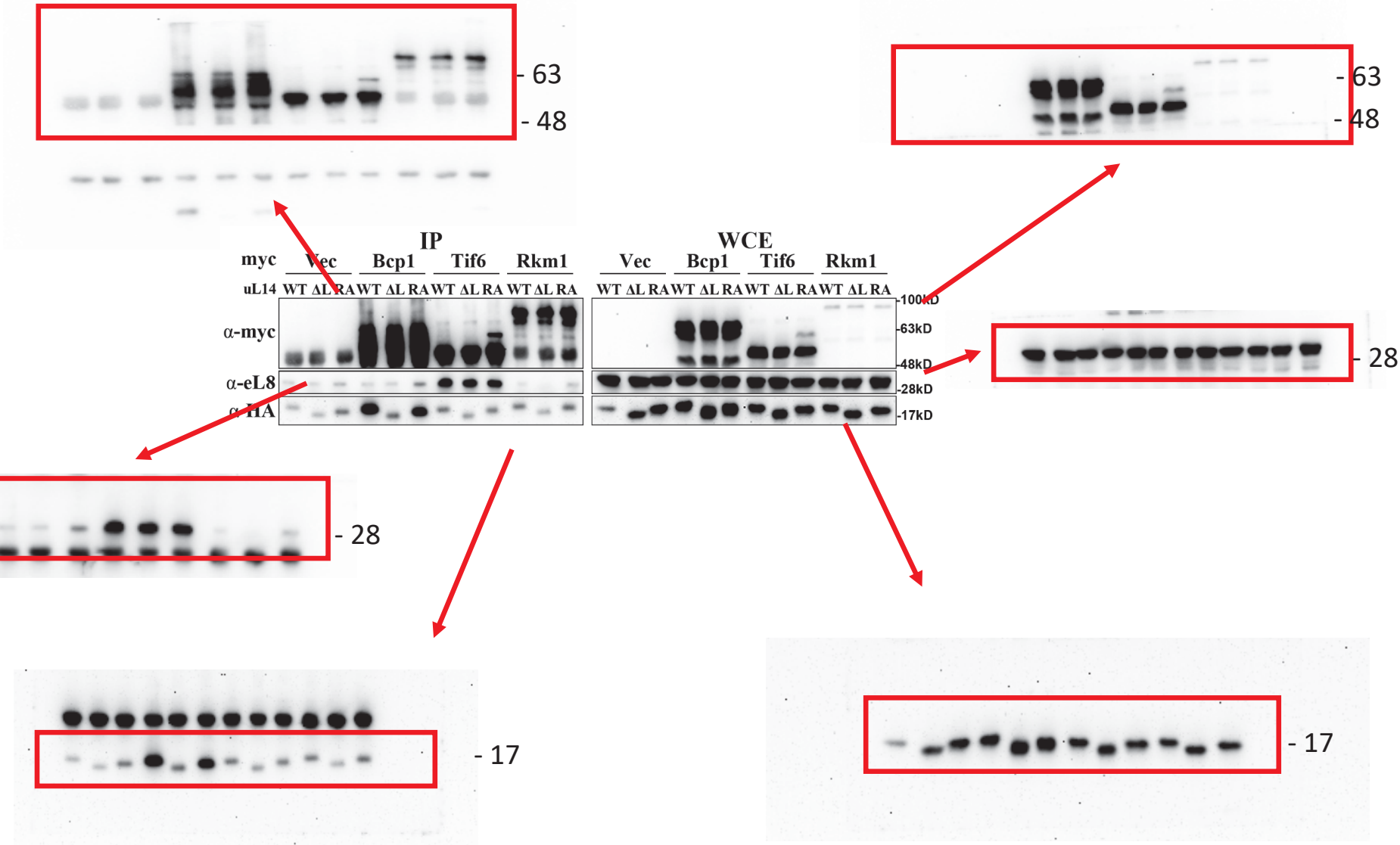

Supplement: SourceData F4 — is the source file for Fig. 4. [file JCB_202306117_SourceDataF4.pdf]

Fig 5E

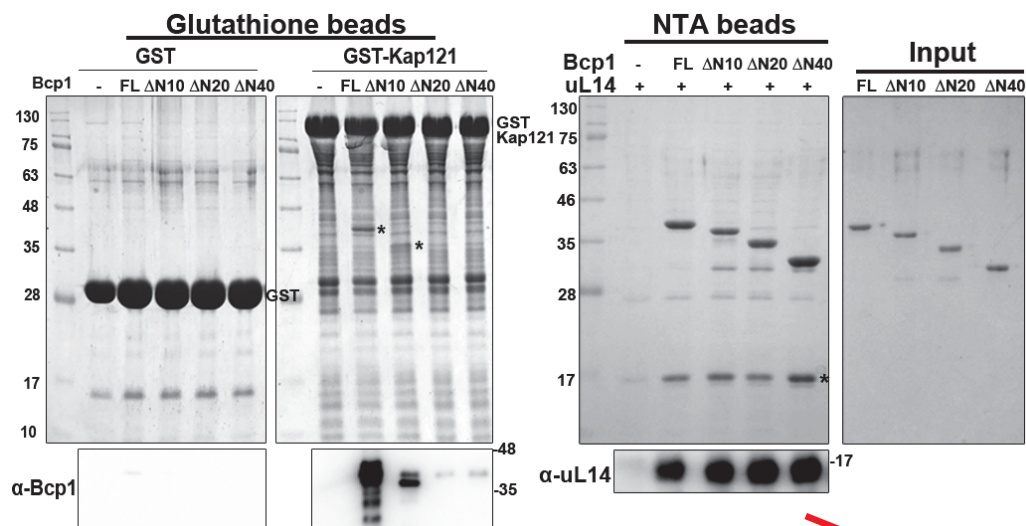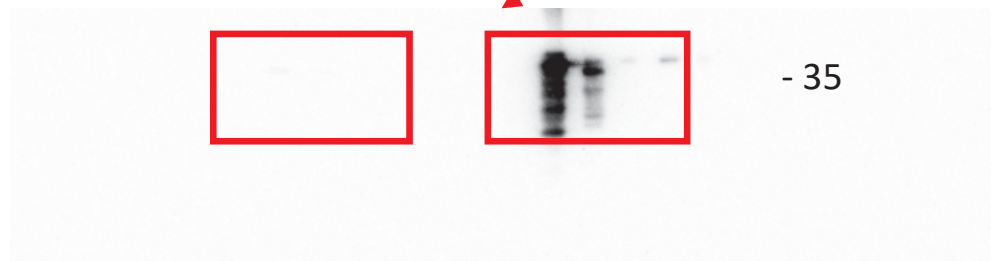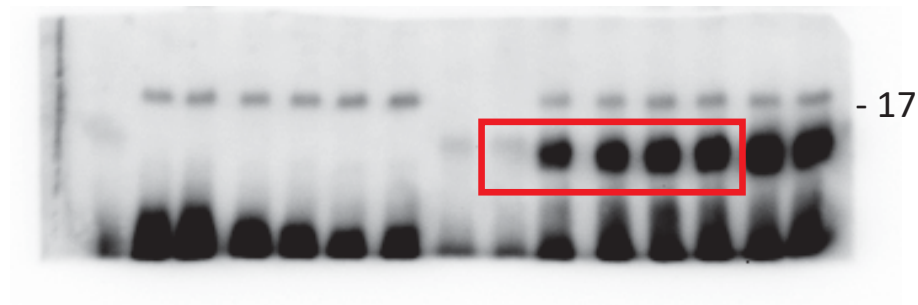

Fig 5F

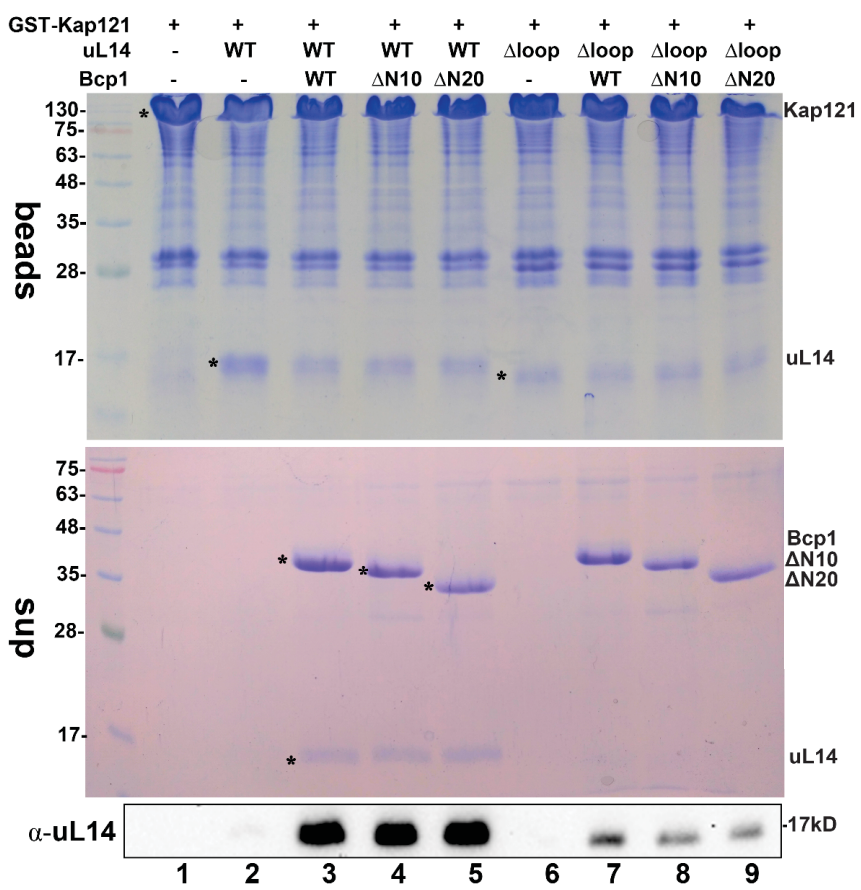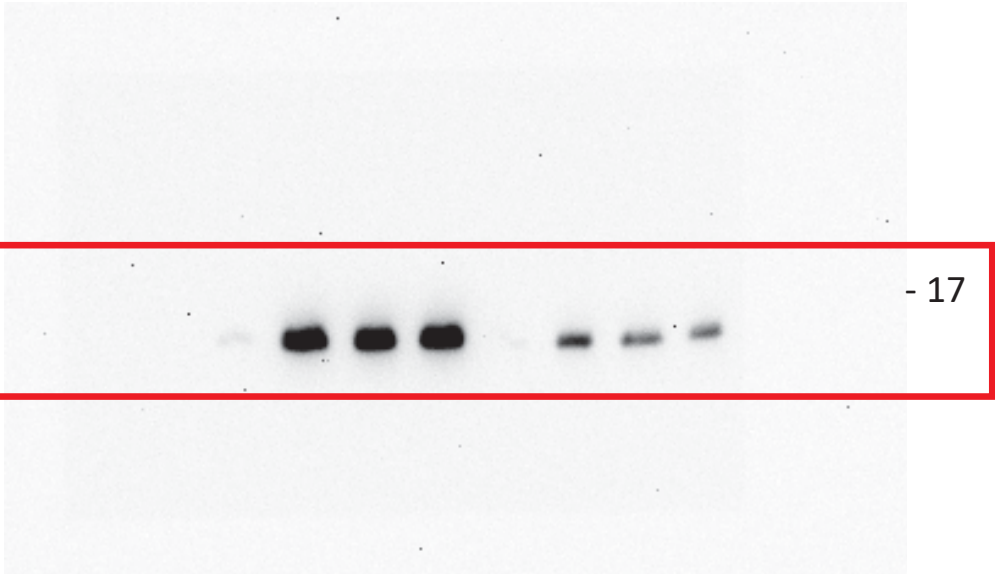

Fig 5G

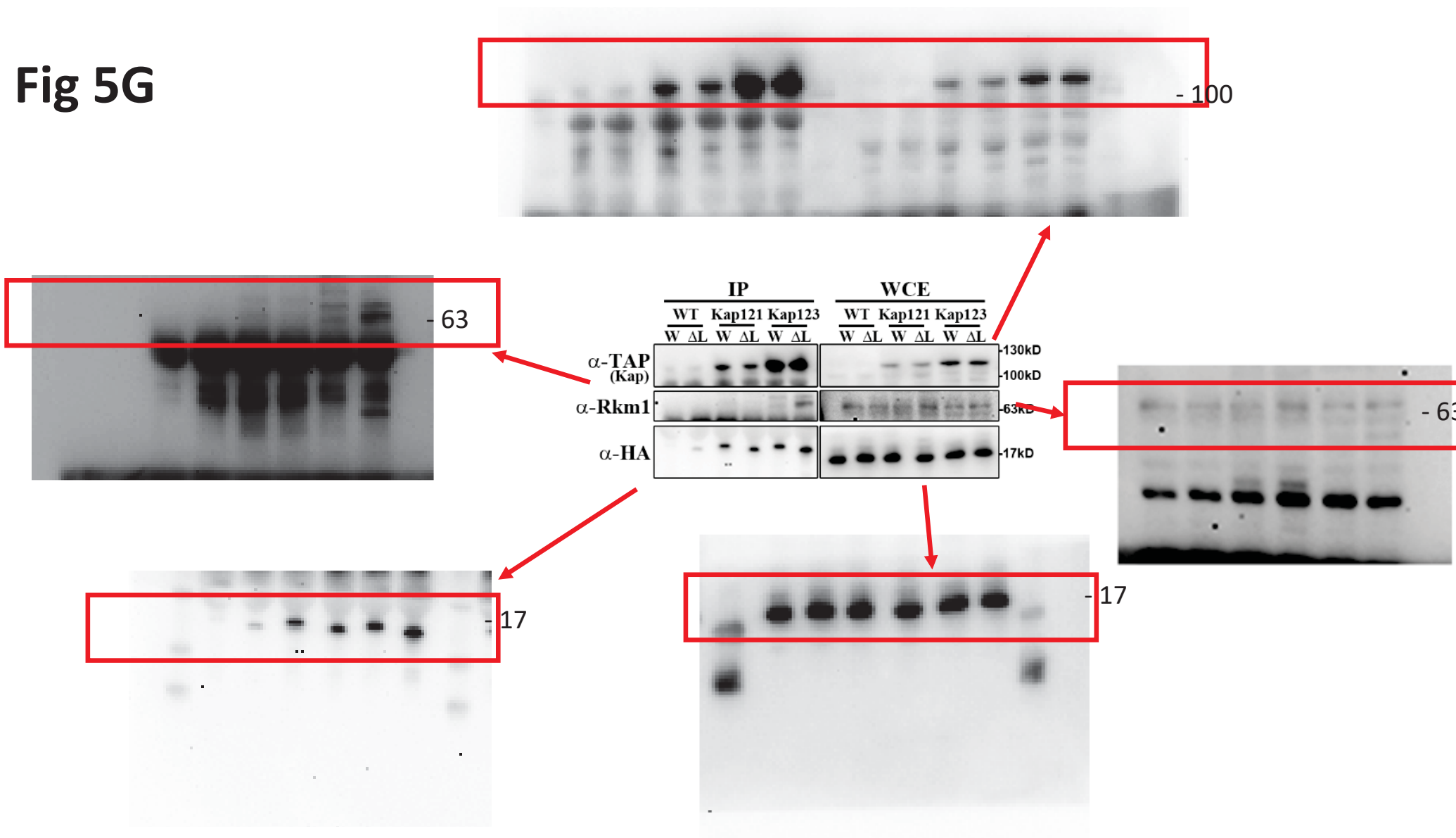

Supplement: SourceData F5 — is the source file for Fig. 5. [file JCB_202306117_SourceDataF5.pdf]

Fig 6A

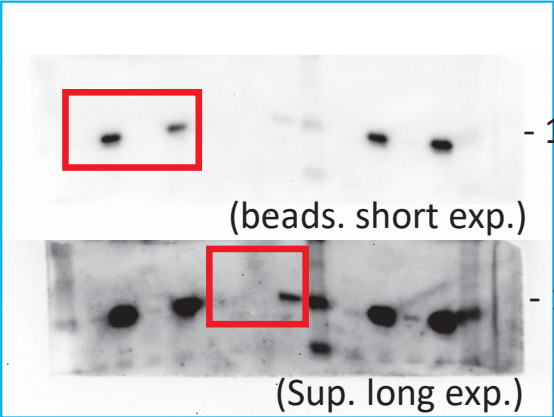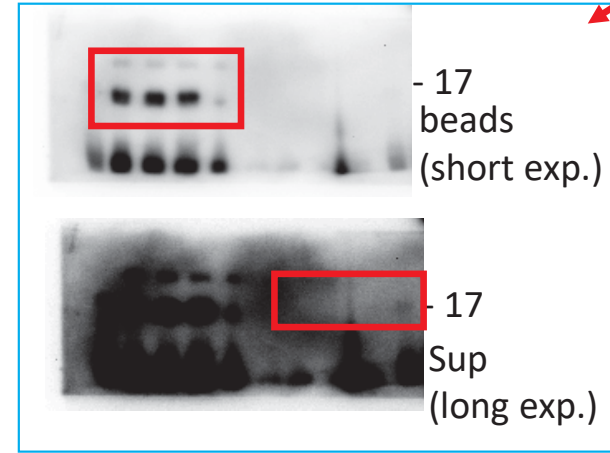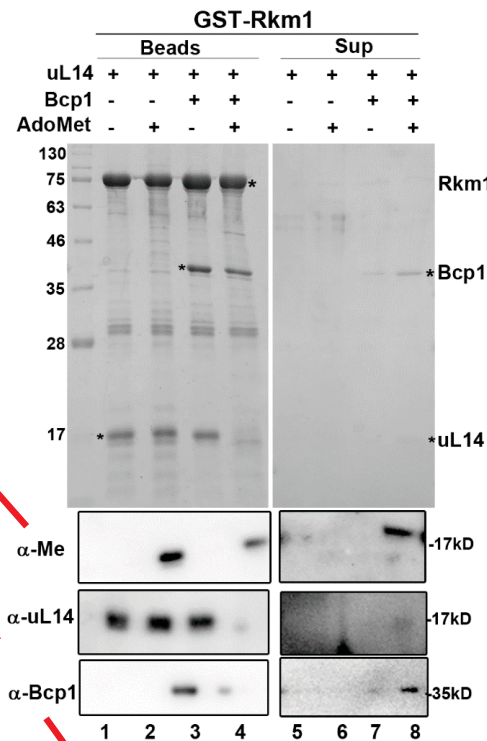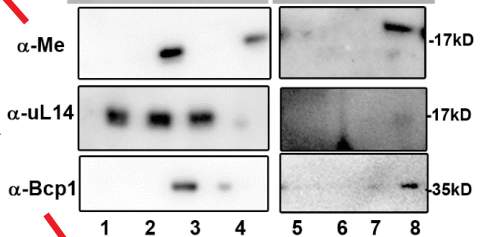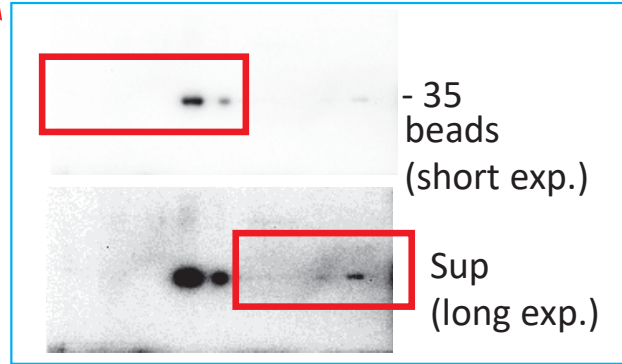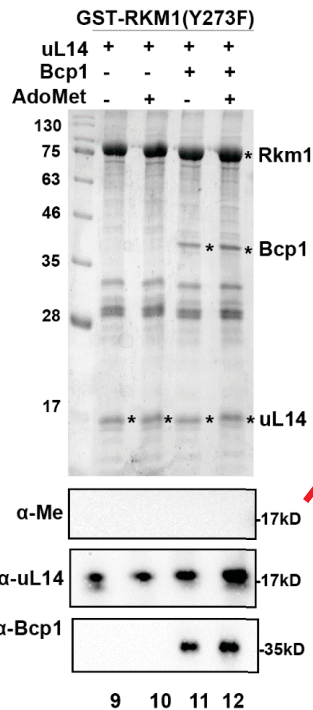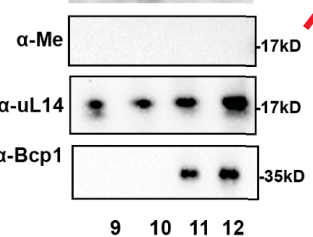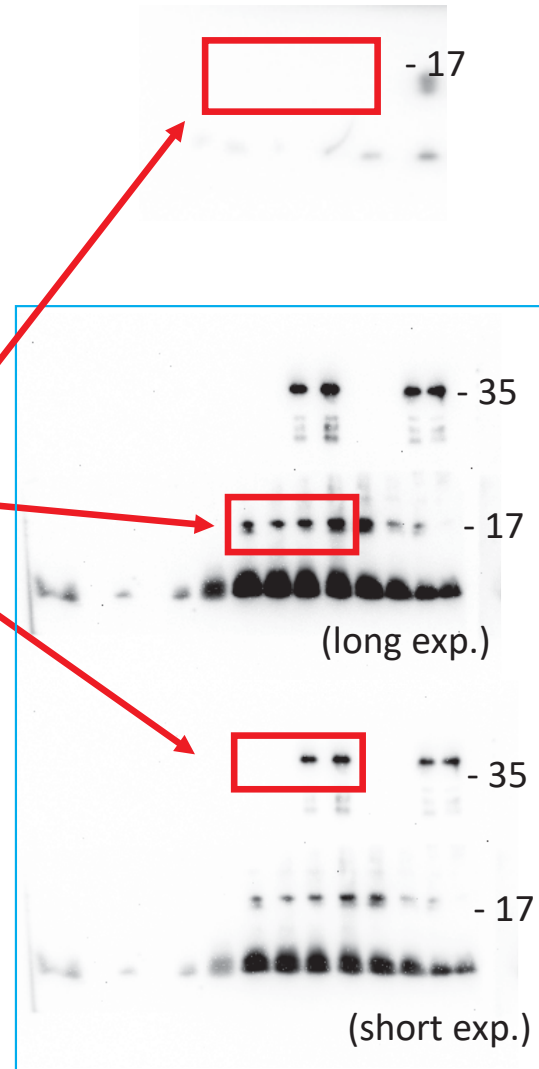

**Fig 6B**

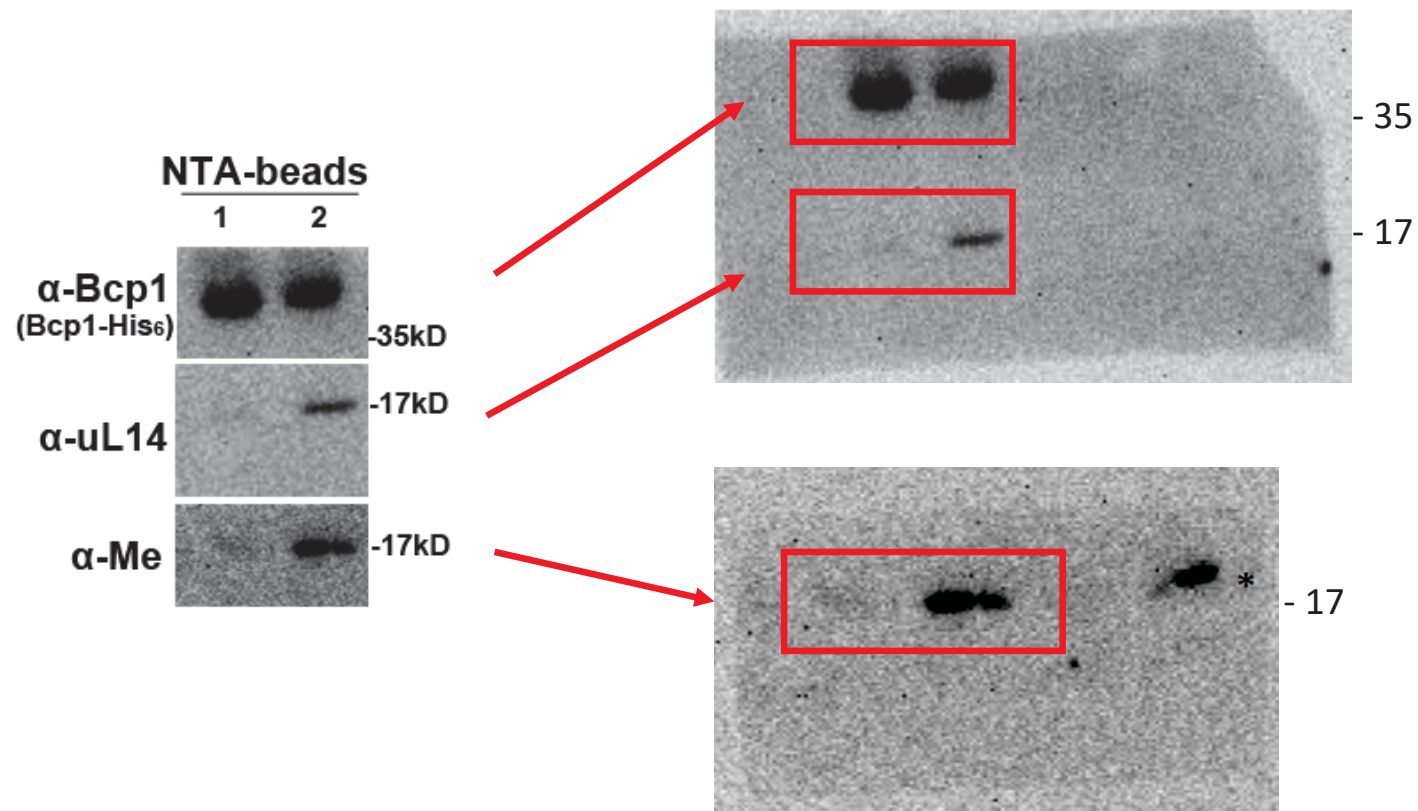

Fig 6D

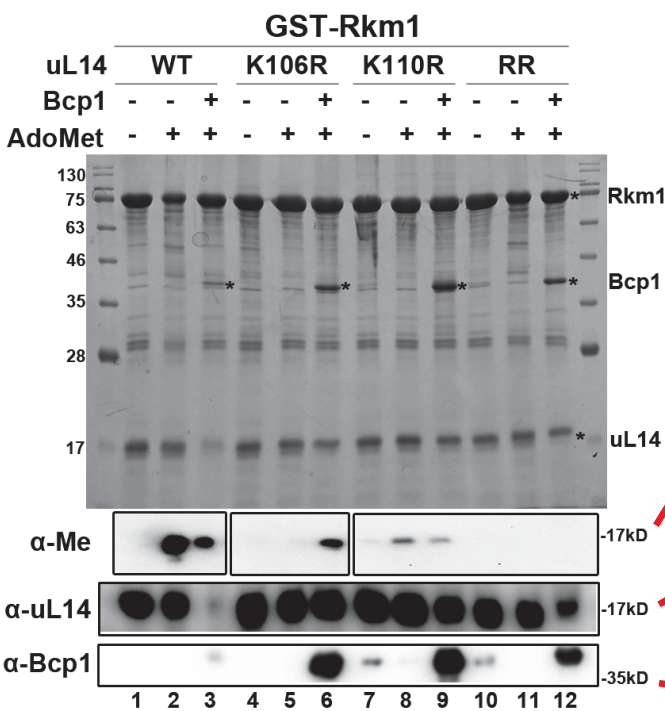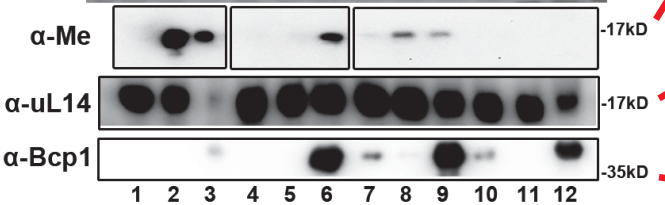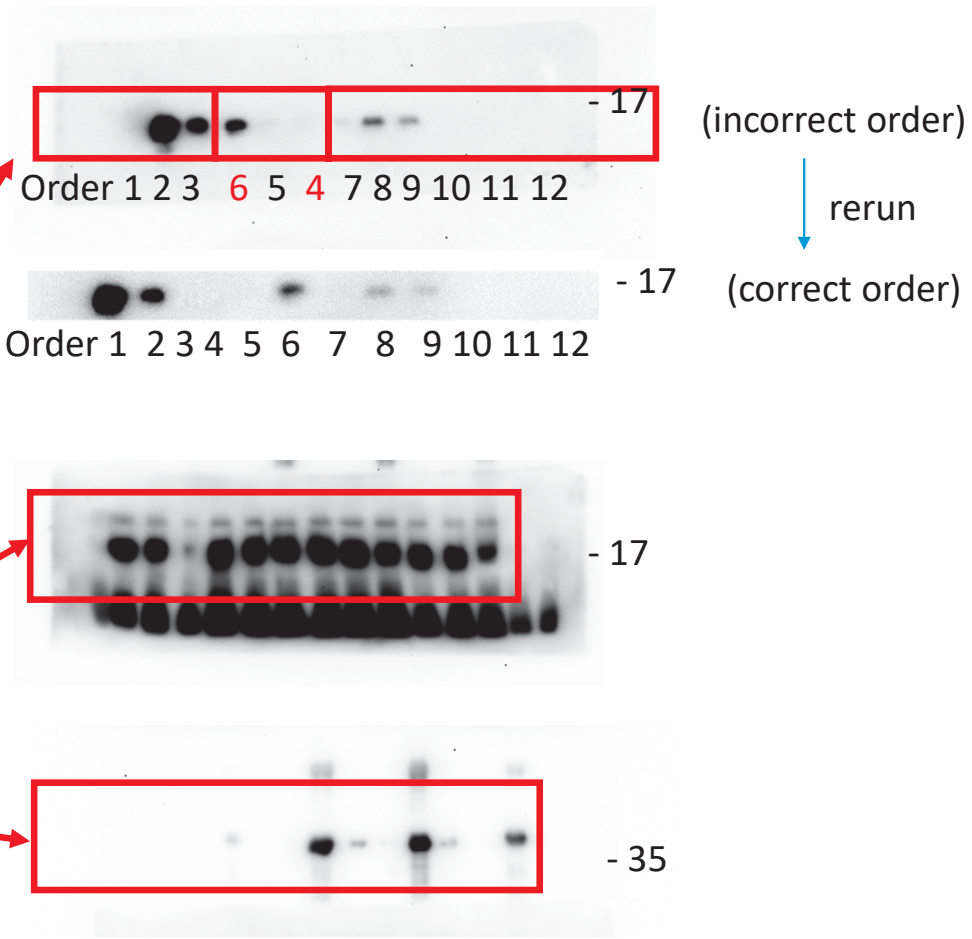

Fig 6E

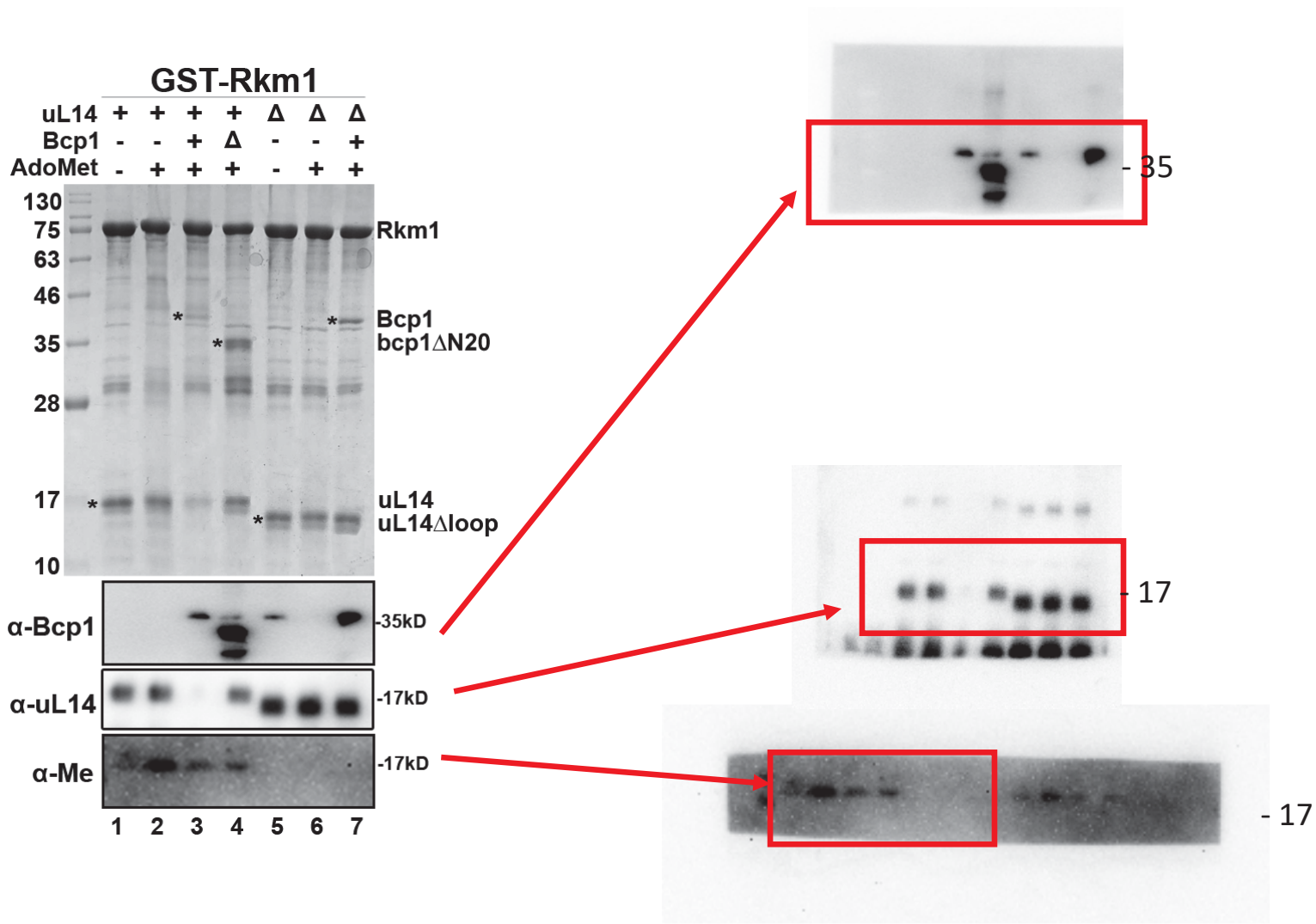

**Fig 6F**

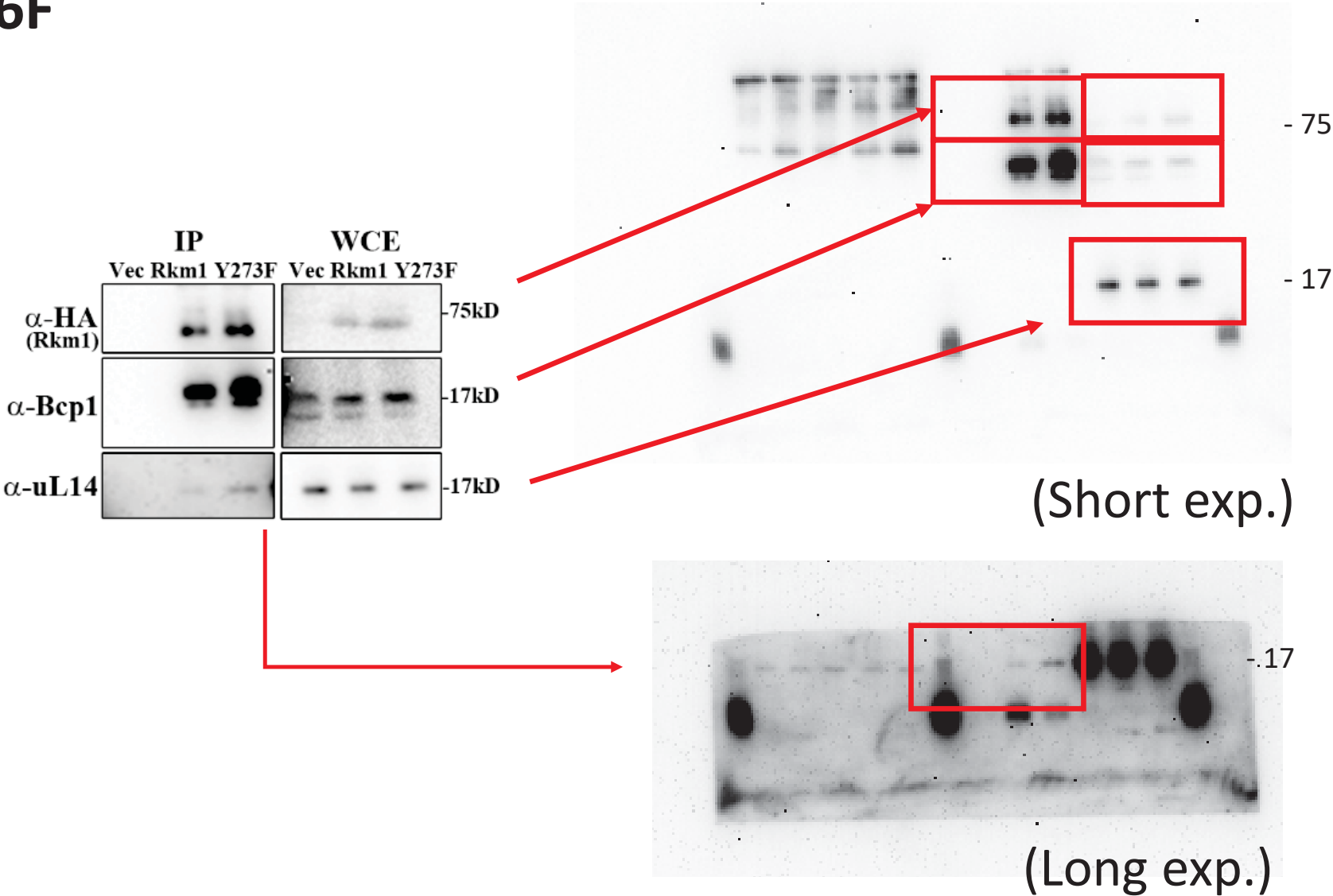

**Fig 6G**

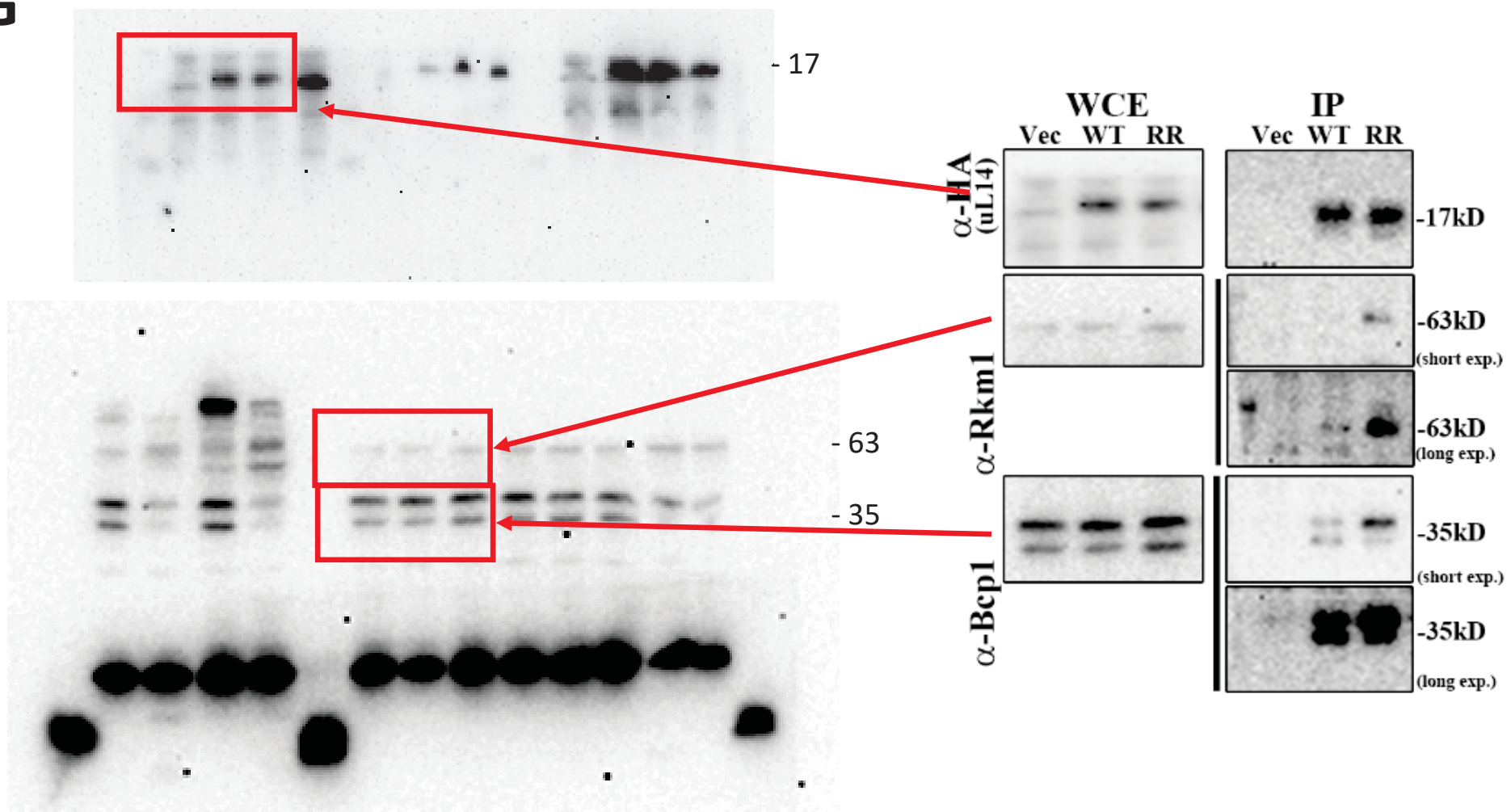

Fig 6G

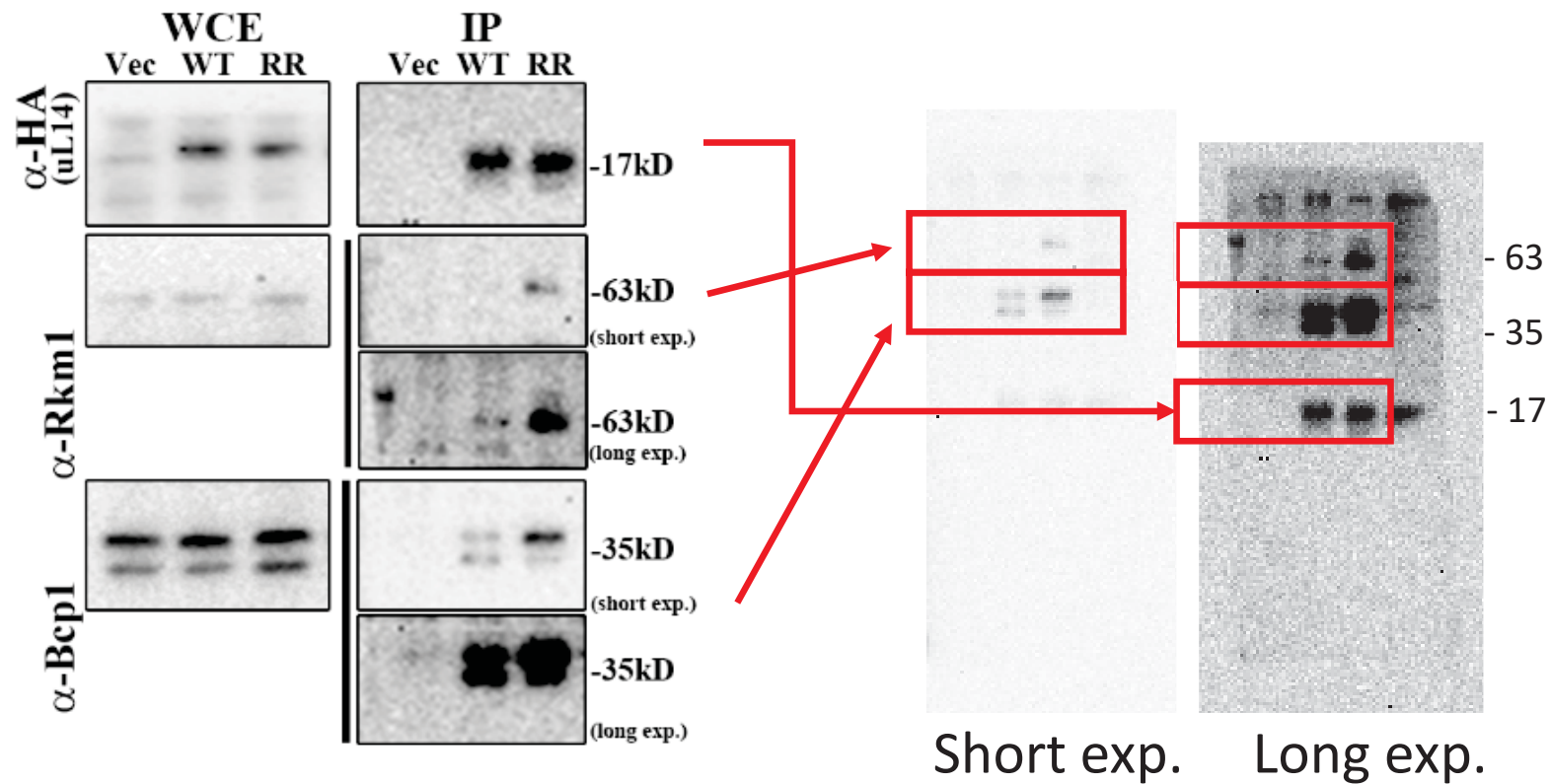

Supplement: SourceData F6 — is the source file for Fig. 6. [file JCB_202306117_SourceDataF6.pdf]

**Fig S1D**

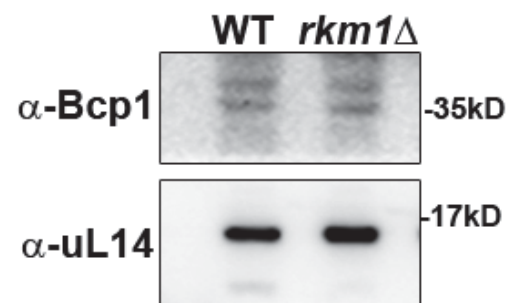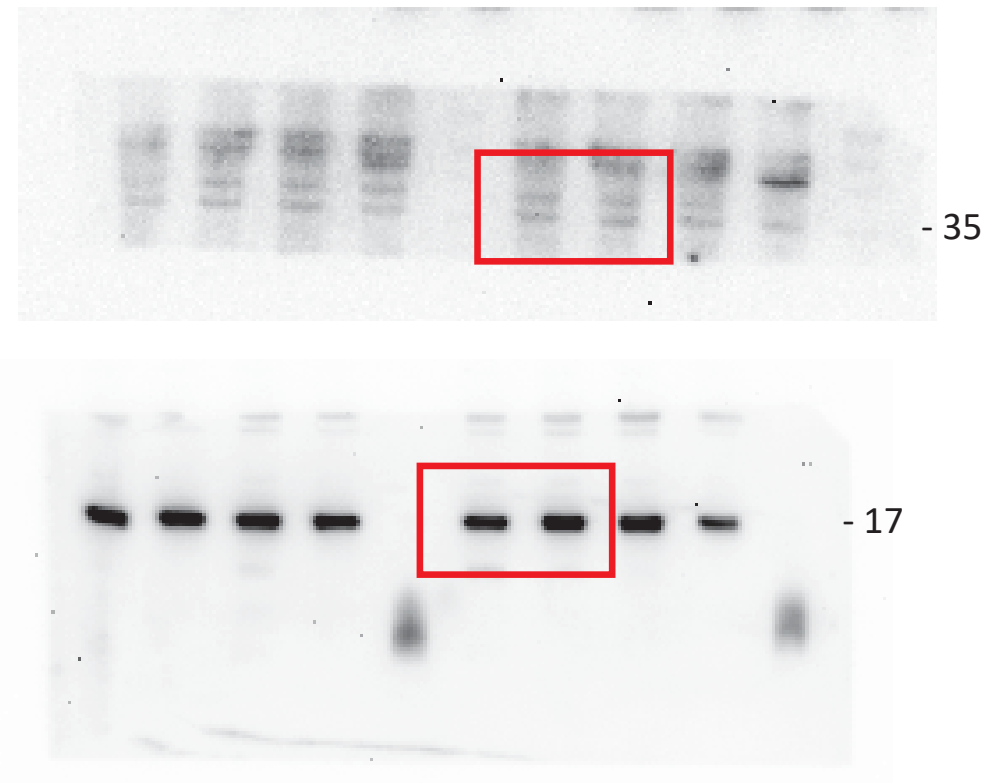

Fig S1E

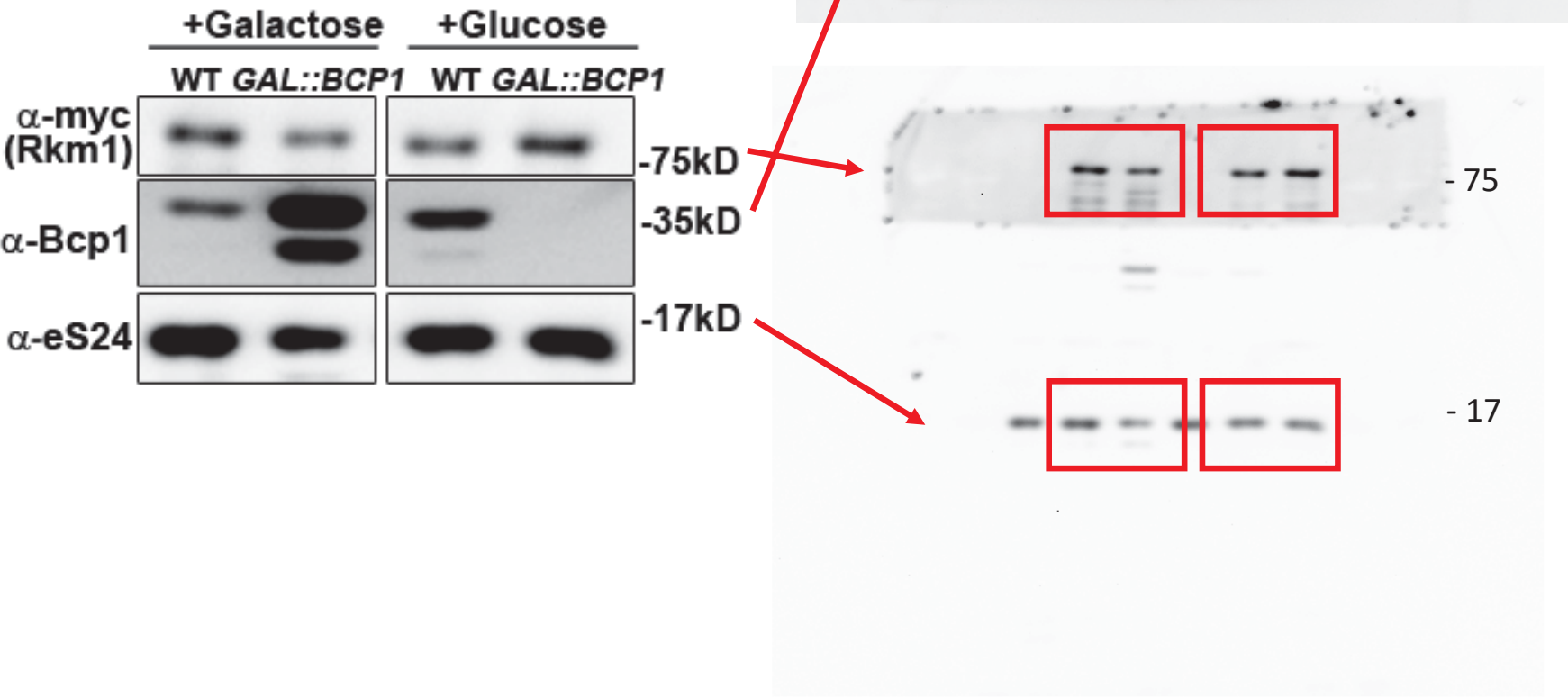

Supplement: SourceData FS1 — is the source file for Fig. S1. [file JCB_202306117_SourceDataFS1.pdf]
